# The Genetic Architecture of Coordinately Evolving Male Wing Pigmentation and Courtship Behavior in *Drosophila elegans* and *Drosophila gunungcola*

**DOI:** 10.1534/g3.114.013037

**Published:** 2014-08-27

**Authors:** Shu-Dan Yeh, John R. True

**Affiliations:** Department of Ecology and Evolution, Stony Brook University, Stony Brook, New York 11794-5245

**Keywords:** pigmentation, courtship behavior, QTL mapping, complex traits, genetics of species differences

## Abstract

Many adaptive phenotypes consist of combinations of simpler traits that act synergistically, such as morphological traits and the behaviors that use those traits. Genetic correlations between components of such combinatorial traits, in the form of pleiotropic or tightly linked genes, can in principle promote the evolution and maintenance of these traits. In the Oriental *Drosophila melanogaster* species group, male wing pigmentation shows phylogenetic correlations with male courtship behavior; species with male-specific apical wing melanin spots also exhibit male visual wing displays, whereas species lacking these spots generally lack the displays. In this study, we investigated the quantitative genetic basis of divergence in male wing spots and displays between *D. elegans*, which possesses both traits, and its sibling species *D. gunungcola*, which lacks them. We found that divergence in wing spot size is determined by at least three quantitative trait loci (QTL) and divergence in courtship score is determined by at least four QTL. On the autosomes, QTL locations for pigmentation and behavior were generally separate, but on the X chromosome two clusters of QTL were found affecting both wing pigmentation and courtship behavior. We also examined the genetic basis of divergence in three components of male courtship, wing display, circling, and body shaking. Each of these showed a distinct genetic architecture, with some QTL mapping to similar positions as QTL for overall courtship score. Pairwise tests for interactions between marker loci revealed evidence of epistasis between putative QTL for wing pigmentation but not those for courtship behavior. The clustering of X-linked QTL for male pigmentation and behavior is consistent with the concerted evolution of these traits and motivates fine-scale mapping studies to elucidate the nature of the contributing genetic factors in these intervals.

The evolution of many complex traits often depends on or is facilitated by functionally related behavioral, physiological, and/or morphological phenotypes. A famous example is the elaborate male fan plumage of peacocks, which seemed so contradictory to natural selection-based explanations that Charles Darwin (1859) proposed a second explanation, sexual selection. The exaggerated plumage and the spread and vibration of the plumage during peacock courtship need to be coordinated for the males to deliver a visual courtship signal to females; otherwise, natural selection would have prevented the fan from evolving. Thus, lack of one of a pair or set of functionally related traits (*e.g.*, a behavior) may render the other (*e.g.*, a morphology) useless or even detrimental to fitness. Such functionally related trait complexes, especially co-evolving morphologies and behaviors, exist throughout nature, for example, in mimetic behavior, diet shifts, habitat adaptation, and courtship behavior (Greene *et al.* 1987; Losos 1990; Grant and Grant 1996; Dewitt *et al.* 1999; Wiens 2000; Marroig and Cheverud 2001; Via and Hawthorne 2002).

Natural selection on multiple traits simultaneously is expected to produce genetic correlations between them through linkage disequilibrium (reviewed by McKinnon and Pierotti 2010). Moreover, quantitative genetic theory predicts that traits that are genetically correlated, either due to pleiotropy or linkage, will evolve in concert (Falconer and Mackay 1996). Many genetic correlations have been documented between morphological traits and functionally related behaviors (Cheverud 1982; Brodie 1989; Cheverud 1996; Marroig and Cheverud 2001; Sih *et al.* 2004; Bell 2005). In theory, linkage disequilibrium lasting longer than a few generations may be due to close physical linkage of underlying loci or strong selection on unlinked loci. Over longer evolutionary times, phenotypic integration of adaptive trait complexes should be promoted by the evolution of genetic integration via selection on pleiotropic loci responsible for the development of multiple component traits (Cheverud 1996; Wagner and Altenberg 1996).

Quantitative trait locus (QTL) studies have provided evidence for shared genetic architecture underlying correlated traits. In a meta-scale analysis of published intraspecific QTL studies, Gardner and Latta (2007) found that approximately one quarter of correlated trait pairs appeared to involve at least one QTL that mapped to the same position. In general, the proportion of shared QTL increased with the magnitude of the genetic correlation, although there was a great deal of scatter around the regression. This analysis was consistent with shared genetic architectures underlying coordinately selected traits, but the resolution of most QTL studies usually cannot distinguish between pleiotropy and close-linkage of loci.

In this study, we addressed whether similar or distinct genetic architecture underlies coordinately evolving components of male courtship display in two Oriental *Drosophila melanogaster* group species, *D. elegans* and *D. gunungcola*. In this species group, various degrees of black pigmentation appear on the apical region of male wings. Kopp and True (2002) reported that the presence of male-specific wing spots is closely phylogenetically correlated with frontal wing display during courtship. Frontal wing displays, coupled with the wing spots, also have been proposed to serve as a visual mating cue in two wing spot−bearing species within the related *suzukii* subgroup (Fuyama 1977, 1979; Hegde *et al.* 2005). Male mating success in the laboratory is affected by the presence of wing spots in *D. elegans* and *D. biarmipes*, two Oriental *melanogaster* group species (S.-D. Yeh, R. Yukilevich, E. Hill-Burns, and J. R. True, unpublished data). *D. elegans*, which possesses both male wing spots and a male frontal wing courtship display, is interfertile with its sibling species, *D. gunungcola*, which lacks both of these characters (Yeh *et al.* 2006). The F_1_ females of this interspecies cross are fertile, providing a powerful system in which to dissect the genetics of these two traits and compare their architectures.

*D. elegans* is widely distributed in southeast Asia with two body color morphs, a dark form in the populations in northern part of the range (Ryukyu Islands and Taiwan) and a brown form in southern populations (Hong Kong, Hainan, Philippines, and Indonesia) (Bock and Wheeler 1972; Hirai and Kimura 1997). In contrast, *D. gunungcola* has only been reported only in mid-high elevation sites in Indonesia (Sultana *et al.* 1999; Suwito *et al.* 2002). As described previously (Kopp and True 2002; Yeh *et al.* 2006), *D. elegans* males exhibit a complex series of actions during courtship, first orienting toward females, circling in front of them while facing them and extending the leading wing 90° outward (“Circling”), then engaging in a frontal two-wing display (“Wing Display”) during which they hold both wings out with ventral sides facing the female while moving their body laterally with the abdomen bended toward the female (“Body Shaking”), followed by tapping the female with their front legs, and finally attempting copulation. In contrast, *D. gunungcola* males perform relatively simple actions, consisting of orienting toward females, tapping them with their front legs, and then attempting copulation.

In our previous study (Yeh *et al.* 2006), we used a small number of molecular markers in backcross progeny to begin to uncover the genetic architectures of these two divergent male traits. We found that both wing spot size and courtship behavior differences are polygenic and that wing spot size is strongly influenced by the X chromosome whereas courtship score shows a smaller X chromosome effect. In a comparative developmental genetic analysis, Prud’homme *et al.* (2006) demonstrated that the X-linked gene *yellow* is expressed in the male wing spot pattern in pupae in *D. elegans* but not in *D. gunungcola*. The *yellow* gene product is an extracellular protein with an as yet-uncharacterized function in dopa melanin formation or sequestration and its late pupal epidermal expression correlates strongly with melanin patterns in diverse *Drosophila* species (Walter *et al.* 1991; Wittkopp *et al.* 2002a,b). The gene expression difference mapped to a handful of nucleotide substitutions in a 775-bp *cis*-regulatory sequence (*spot^ele^*) located in an anciently evolved wing regulatory element present throughout *Drosophila*.

In the work reported here, we used 41 molecular markers to perform a genome-wide QTL mapping analysis in backcross populations of the *D. elegans* × *D. gunungcola* hybrid cross, providing the first comprehensive mapping of the genetic architecture of coordinately evolving male wing pigmentation and courtship display traits. At least five QTL were found for both wing spot size and courtship score. Importantly, two regions of the X were associated with QTL clusters for both wing spots and courtship, one of which contains the *yellow* gene. We also performed a genome-wide test of epistasis underlying wing spot size and courtship scores, as well as individual courtship elements.

## Materials and Methods

### *Drosophila* strains, cultures, and karyotyping

*Drosophila elegans HK* and *D. gunungcola* SK originated from several females collected in Hong Kong, China, and Sukarami, Indonesia, respectively (Ishii *et al.* 2002). These flies were kindly provided by Dr. M.T. Kimura and maintained on standard fly media (molasses, corn meal, yeast extract, and agar) in a 25° incubator. The karyotyping procedure was described by Sullivan *et al.* (2000).

### *Drosophila* crosses

The crosses used in this study have been described previously (Yeh *et al.* 2006). In summary, reciprocal interspecific crosses were performed by keeping 5−10 virgin females from one species and males from the other species in a fresh vial and transferring them into a new vial every week for several weeks. F_1_ hybrid females were backcrossed to males of either parental species. The difficulty of producing F_1_ and backcross progeny necessitated the use of four types of backcross progeny for QTL mapping analysis. These were: backcross progeny with *D. elegans HK* grand-maternity and paternity, backcross progeny with *D. gunungcola SK* grand-maternity and *D. elegans HK* paternity, backcross progeny with *D. elegans HK* grand-maternity and *D. gunungcola SK* paternity, and backcross progeny with *D. gunungcola SK* grand-maternity and paternity. Of the 152 individuals with *D. elegans* paternity, 93 were progeny of F_1_ females from the original cross *D. elegans HK* females × *D. gunungcola SK* males and thus possessed *D. elegans* cytoplasm. The remaining 59 were progeny of F_1_ females from the reciprocal cross and thus had *D. gunungcola* cytoplasm. Of the 111 individuals with *D. gunungcola* paternity, 72 were progeny of F_1_ females from the original cross *D. elegans HK* females × *D. gunungcola SK* males and thus possessed *D. elegans* cytoplasm. The remaining 39 were progeny of F_1_ females from the reciprocal cross and thus had *D. gunungcola* cytoplasm.

We separately analyzed the effect of cytoplasm on each trait in each backcross. We found no significant effects of cytoplasm on any of the traits in either backross (Mann-Whitney two-sample test, normal approximation; *ele* backcross: wing spot size: S = 3663.5, Z = 0.928, *P* = 0.353, courtship score: S = 4045.0, Z=-0.134, *P* = 0.894; *gun* backcross: wing spot size: S = 1720, Z = 0.028, *P* = 0.978, courtship score: S = 2079, Z = 0.082, *P* = 0.934). Therefore, we pooled the backcross progeny with *D. elegans* paternity and refer to these as *elegans* (*ele*) backcross progeny. We also pooled the backcross progeny with *D. gunungcola* paternity and refer to these as *gunungcola* (*gun*) backcross progeny. In total, 152 and 111 males from the *ele* backcross set and the *gun* backcross set, respectively, were phenotyped and genotyped for the QTL mapping analyses. Potential caveats of pooling across cytoplasmic backgrounds for linkage mapping are examined in the *Discussion* section.

### Behavioral scoring of males

Virgin females from the parental species and male backcross progeny were collected under light CO_2_ anesthetization and aged in food vials for 3−5 d in groups of 5−20 after eclosion in a 25° incubator with a 12:12 light:dark cycle. At least 24 hr before the observation of courtship behavior, males were anesthetized lightly with CO_2_ and individually separated into food vials. In the courtship behavior assay, one 3- to 5-d-old virgin female from each species was transferred into a food vial containing one male backcross progeny. The presence or absence of three courtship elements, wing display, circling, and body shaking (Yeh *et al.* 2006) were recorded until copulation occurred or 1 hr elapsed. Observations of courtship behavior were repeated the next day by adding one additional 3- to 5-d-old virgin female from each species. In between the trials the males were kept with the original two females and these females were present for the second trial. During both the observation periods, the presence or absence of three courtship elements: wing display, circling, and body shaking (Yeh *et al.* 2006) were recorded until copulation occurred or 1 hr elapsed. Male backcross progeny then were assigned a courtship score (ranging from 1 to 4) based on the courtship elements they performed (Yeh *et al.* 2006).

For the individual courtship elements, the presence of the behavior in either of the two trials was taken as evidence of the presence of the element. For two-wing-display analysis, males were categorized into two groups: (1) those exhibiting steady wing extensions or (2) those exhibiting any other types of wing movements or no wing movement at all. An alternate binary analysis in which males were classified as either having or not having any two-wing movements did not yield any significant QTL. For the circling element analysis, males were categorized into three states: (1) those that moved to the head-to-head position, (2) those that moved only to the side of the female, or (3) no circling. For the body shaking element analysis, four categories were distinguished: (1) vigorous body shaking, like *D. elegans* males, (2) slow body shaking, (3) subtle body shaking, as occasionally exhibited by *D. gunungcola* males, or (4) no body shaking, like *D. gunungcola* males. This courtship element was recorded independently of wing display, although wing movement sometimes makes the body movement more conspicuous. After the courtship assays, males were preserved in 1.5-mL microcentrifuge tubes individually at −20°.

### Wing spot size measurements

The right wings of male backcross progeny were mounted in glycerol with 10% ethanol on glass slides and photographed with a Zeiss Axiocam HRC digital camera under a Leica MZ7.5 dissecting microscope connected to a Dell PC using Zeiss AxioVision (Rel 4.3) software. The entire wing dataset was imaged on the same day with the same settings. Wing spot size, which is the area with visible melanin, was measured in Image J 1.31v software by two different workers. Each wing spot size measure was divided by wing area (wing length × wing width). Our proxy for wing length was the length of the line between the intersection of the anterior crossvein and the L4 vein and the intersection of the L1 and L3 veins on the wing margin. Our proxy for wing width was the length of the line between the intersection of the L1 and L2 veins on the wing margin and the intersection of the L5 vein and the wing margin. These standardized values obtained from the two different workers were then averaged for use in QTL analysis.

### Molecular marker genotyping

Genomic DNA was isolated using the single fly preparation protocol described previously (Yeh *et al.* 2006). Single fly genomic DNA of parental species was used to acquire the DNA sequences of marker loci, and these sequences were checked for allelic monomorphism within the parental species/strains and differences between species were noted. We chose a set of 41 loci covering approximately the entire genome (Table 1). Many of these genes are candidate genes for either behavior or pigmentation based on their functions in *D. melanogaster*. For initial sequencing of *D. elegans HK* and *D. gunungcola SK*, primer sequences were designed based on conserved regions among the genomes of the *D. melanogaster* species subgroup, *D. ananassae*, and *D. pseudoobscura*. The specific primers for genotype diagnosis in backcross progeny were then designed based on the DNA sequence of *D. elegans HK* and *D. gunungcola SK*. These primers are listed in Table 1. Before examining the genotype of each locus in backcross progeny, the allelic difference between species and lack of allelic variation within species were confirmed by genotyping the marker in five females and four males from each species.

**Table 1:** Description of molecular markers used in this study

Locus	Abbreviation	Cytological Position*^a^*	Primer Sequence (5′ to 3′)	T_A_*^b^*, °	Allelic Difference Between *D. elegans* HK and *D. gungungcola* SK
X					
* yellow*	*y*	1A5	CCCAGCCCATACCCTTTCAAAAATG	60	Indel: 43 bp deletion in *D. elegans* HK
			AATCCTCTTCTGTGGACCGTGGCGCGC		
CG2658	CG2658	3E1-2	AGCTCCTGGTCGAGATGGATGG	60	Indel: 2 bp deletion in *D. gunungcola* SK
			AGGATGTGGCGATCGAAGCGAC		
* Tyramine beta hydroxylase*	*Tbh*	7D2	AGCAGGACTGCGAGGTCTTC	60	Indel: 7 bp deletion in *D. elegans* HK
			TACATGGTGCCCTCCTGGAA		
*Moesin*	*Moe*	8B4-6	CCGKAAYACATTCAAGTATGG	56	Indel: 121 bp deletion in *D. elegans* HK
			AGATCCTGTTTCAGGGCCTGAA		
* tan*	*t*	8D1	TTGTTCGTACATATCAAATATGCCTT	54	Indel: 9 bp deletion in *D. gunungcola* SK
			ACATACGCAATATAAGTGCTTTACAC		
* dusky*	*dy*	10E2	AAGGGATTCCATWAGAYTCCATACTACAG	58	Indel: two alleles, about 150 and 250 bp insertion in *D. gunungcola* SK(a)
			AAGAGCACATCACAATGGATTAAGG		
* cacophony*	*cac*	10F8-11A1	ATCCTCGCCTTAGGGCTTGTTCTG	54	Indel: 6 bp deletion in *D. elegans* HK
			TTGCACGGCGTGGTTGACAT		
* upheld*	*up*	12A7	CATCGTAYCMATCGATTTCC	54	Indel: 8 bp deletion in *D. gunungcola* SK
			TACGTGCGGTACAAACGTGG		
* small wing*	*sl*	14B15-17	AACGTGCAGAGGGACAACTCG	64	Indel: about 200 bp deletion in *D. elegans* HK
			CGAATATCTCAATGGAGTCGGTTC		
* courtless*	*crl*	14F1	CCTGTTCACTTGCCCATTGATCTTG	54	Indel: two alleles, 17 and 23 bp deletions in *D. elegans* HK (b)
			ACTCTCATCGTTGGGTTCTGTTGG		
* outstretched*	*os*	17A5	GCAACTGGATCGACTATCGCAACT	54	Indel: two alleles, 14 and 20 bp insertions in *D. elegans* HK(b)
			ACTTACCGTGGAATTGGGCTTGAG		
CG11943	CG11943	18F4	ACTCGGAGAGCATTGTGAACACG	56	Indel: 12 bp deletion in *D. elegans* HK
			TGTCGCCCGGTCTCGTGTAG		
2L					
* aristaless*	*al*	21C3	GAGAATTCAGGGGCTCCAAGCTG	66	Indel: 15 bp deletion in *D. gunungcola* SK
			AACTGACCGGGCATGTAATGAC		
* timeless*	*tim*	23F6	ATGGCGACTACGAGGATCAG	62	Indel: about 400bp deletion in *D. elegans* HK (a)
			TTCAAGGGTTCAGAGGCTGC		
* echinoid*	*ed*	24D4-6	ATGGAGCTCACATGCAGCAG	62	Indel: 4 bp deletion in *D. gunungcola* SK
			ATTGGTGGGCTGATCCTTGG		
* arylalkylamine N-acetyltransferase 2*	*aaNAT2*	26C1	ACGAGCCGCTGATGCTGATCC	58	RFLP: *Hph I*
			CTGTCCACGCCGAGCATGTAG		
* Btk family kinase at 29A*	*Btk29A*	29A1-3	CCACAGTCGCATGTGAAGCACTA	54	RFLP: *Alu I*
			CCATGCTTCATGTACTCGGTGAC		
* black*	*b*	34D1-3	ATCTCGGAGCTGTGCAAGAAG	62	Indel: 2 bp deletion in *D. gunungcola* SK
			CCACAGTCGCATGTGAAGCACTA		
* yellow c*	*yellwow_c*	35B8	TGGAGCGCTGGCAAACAGAATC	58	Indel: 7 bp deletion in *D. gunungcola* SK
			GGTTTCCACTCTTGACGCTCGATG		
* Dopa decarbosylase*	*Ddc*	37C1	AAGGCAGTTTAAGCGACCTTCC	54	Indel: 39 bp deletion in *D. elegans* HK
			TCAGATACCCGGGCTTCACTTC		
2R					
* Ecdysone Receptor*	*EcR*	42A6-9	AGAGGATCTCAGGCGTATAATG	56	RFLP: *Mlu I*
			CMGCCATTCCGGCCATTTTGTA		
* spinster*	*spin*	52E5-8	CAGAACAATAACAACCCGTACAATGG	58	Indel: two alleles, 26 and 31 bp insertions in *D. elegans* HK (b)
			TGACGATGACAATCTCCAAGTGC		
* Dopamine Transporter*	*DAT*	53C7-8	ATTGAAGTCCCTGGATTGCT	54	Indel: 6 bp deletion in *D. elegans* HK
			AATATCATCACAACCCGTTCG		
* Black cells*	*Bc*	54F6	ATCTGCCCGAAAGTGGATGAG	57	Indel: 296 bp deletion in *D. elegans* HK
			GGTTTCGCACTCTTGACATTCC		
* Dopamine N acetyltransferase*	*aaNAT1*	60B12-C1	CAAGGCGGTCAACAAGAAGG	60	Indel: 117 bp deletion in *D. gunungcola* SK
			ACATCATCGGGGGACTGTTG		
3L					
* bric a brac 1*	*bab1*	61E2-F1	CTAAATCGCAGCATTGGTCTTAC	46	Indel: 11 bp deletion in *D. gunungcola* SK
			TCACTTGTATTGTAAGGCAGGGA		
* yellow g*	*y-g*	62D5	CCCAARATCGTKGCCATMAACAC	57	Indel: about 400bp deletion in *D. gunungcola* SK (a)
			TTGTTKCCSGTGATGTCGTANAC		
* pale*	*ple*	65C3	AAGCTCTTTGTCCCAGCCTAATTGC	56	Indel: 12 bp deletion in *D. elegans* HK
			GCAGAGGAGAACGAACGCTTGTT		
* Clock*	*Clk*	66A12	CCGCAATTCTGAAACAAAACAAC	54	Indel: 135 bp deletion in *D. elegans* HK
			TATGCGGAGTCTTGGGATTATTG		
* aracaun*	*ara*	69C8-69C10	GYGAGAAGATYATGCTGGCCAT	55	RFLP: *ScrF I*
			ATGGCATCCTCCTCCTCTTTGG		
* yellow k*	*yellow_k*	71D4	ACTCTGATCGAAGCGCAGTG	60	RFLP: *Dra II*
			CCAGTCTATCCACGGTGCTG		
* Baldspot*	*Baldspot*	73B4-5	GCAAAGCAAGAACTAGTCAATAGCAC	59.5	Indel: 56 bp deletion in *D. elegans* HK
			CTGGTGTATTCCGTGTAGCTG		
3R					
* yellow e*	*yellow_e*	87E10	CAGCCCTTGGCCACTGATAG	54	Indel: 23 bp deletion in *D. gunungcola* SK
			AACCAACATTAACCTGGCATTGAA		
* Dopamine Receptor (1)*	*DopR*	88A10-12	TCAGTTTCTACTTCCCCTGTGTGG	58	Indel: two alleles, 19 bp deletion or 2 bp insertion in *D. gunungcola* SK (b)
			GAAGAGATTGCATTTATGCCTCCAG		
* fruitless*	*fru*	91A7-B3	GGAGAGAGGGTAAAGGGGATATAG	54	Indel: 4 bp deletion in *D. elegans* HK
			TAGAACGGAAAAGGGTTACAGG		
* Hairless*	*H*	92F3	TAACACATGGGACTCCGGTTC	58	Indel: 27 bp deletion in *D. elegans* HK
			GAGCTGTTGTCATCCGAAACTG		
* ebony*	*e*	93C7-93D1	AAGTGCATGCAGGCGATGTTCTCG	57	RFLP: *Sca I*
			GGTGGCAGTAACCAGACTTGATTCT		
* torso-like*	*tsl*	93F9-10	AAGGAGCCCACGAGGAACATTTAC	56	Indel: 10 bp deletion in *D. gunungcola* SK
			TTTCSCCACAATCTGGTTARCCAG		
* Transcription factor II A-L*	*TFIIA-L*	97E11-97E11	CGCATTCTTGTGCCATTTGTATG	56	RFLP: *Ms I*
			ATGGCTTTACCTTGGTGCTCTG		
* Dopamine Receptor2*	*DopR2*	99B5-6	TCATCCGCAGCCACTGACAT	59	Indel: 16 bp delection in *D. gunungcola* SK
			ACTCATACGCCTTGTAGCCACAT		
4					
* cubitus interruptus*	*ci*	102A1-A3	GTATTCGTGCCAGCATTAGC	60	RFLP: *Alu I*
			CTGTAAACGTGGTGCCAATG		

a Cytological position in *D. melanogaster* is obtained from Flybase (http://flybase.org/).

b PCR program, 1 cycle at 94° 5 sec, 40 cycles at 94° 30 sec, T_A_ 30 sec, 72° 20−70 sec, 1 cycle at 72° 3 min.

One of three diagnostic methods was applied to determine marker genotypes of backcross progeny, depending on the DNA sequence difference between parental species. (1) Genotypes were examined by running horizontal agarose gel electrophoresis directly after target fragments were amplified if a large (>20 bp) insertion/deletion existed between species. (2) Genotypes were determined using an ABI 3130 capillary autosequencer (ABI3130) and analyzed in ABI GeneMapper (Version 3.7) software after target fragments were amplified with fluorescent-labeled primers. This method was used when small insertions/deletions could not be distinguished by horizontal gel electrophoresis. (3) Genotypes were determined by running horizontal agarose gel electrophoresis after amplified fragments were incubated with species-diagnostic restriction enzymes. dCAPS Finder 2.0 (Neff *et al.* 2002), was used to search for diagnostic restriction sites. This method was used when the two species exhibited single nucleotide polymorphisms but not insertion-deletion differences. PCR amplifications were run as follows: 95° 5 min to denature genomic DNA, then 40 cycles in 95° 30 sec, annealing temp 30 sec, and 72° from 20 sec to 1 min, 30 sec depending on the amplified fragment length (see Table 1 for the specific method used for each marker). DNA sequencing and fragment analysis were carried out in the MEAD Laboratory in the Stony Brook University Department of Ecology and Evolution. The GenBank accession numbers of DNA sequences that were used to design the markers for genotype diagnosis are listed in Supporting Information, Table S1.

### Linkage mapping

*D. elegans/D. gunungcola* hybrid linkage maps were constructed separately for the *elegans* and *gunungcola* backcrosses and QTL analysis of each backcross used the linkage map specific to that backcross. Linkage mapping was performed using MapMaker 3.0 (Lander *et al.* 1987). Linkage groups were determined by using the “group” command with a threshold of LOD (logarithm of odds) 3 and 50 cM maximum distance. The relative positions of markers were explored by multiple point analysis and the Haldane map function was employed to compute the genetic distances.

### QTL analysis

Two statistical methods, interval mapping (IM; Zeng 1993) and composite interval mapping (CIM; Zeng 1993, 1994) were used in our QTL analysis. We used IM for preliminary mapping of continuous traits and preliminary and final mapping of binary traits, such as Spot Presence and the individual courtship elements. IM, a likelihood ratio test method, tests the likelihood of QTL regions by using one marker interval at time to construct a putative QTL and testing every position in the interval (Lander and Botstein 1989). The genome scan interval, or walk speed, was 1 cM in our analyses, because a walk speed <1 cM in the IM method was beyond the memory capacity of our computer. It has been reported that the IM method may be biased when multiple QTL are located in the same linkage group (Zeng 1993). Therefore, we used CIM to refine the QTL region of the continuous traits Spot Size 1, Spot Size 2, and Courtship Score. CIM tests the significance of candidate QTL by combining IM with multiple regression analysis. When the putative QTL in an interval is tested, the rest of markers can be used as covariates to control for QTL other than the putative one. By doing this, the residual variance is reduced (Kao *et al.* 1999). We chose five random markers as controls, a 10-cM window size as the control range, and 0.5-cM walk speed for our CIM analyses. The threshold values of likelihood ratio (LR) statistics for testing QTL were determined by permutation tests (Doerge and Churchill 1996). In each analysis, the data were permuted 1000 times, and the 50th largest LR value was set to the experiment-wise significance level of *P* = 0.05. IM and CIM QTL analyses were performed using Windows QTL cartographer 2.5 (Wang *et al.* 2005). Multiple interval mapping, which is proposed to improve the resolution of QTL statistics (Jiang and Zeng 1995; Kao *et al.* 1999), showed very little statistical power for our datasets, possibly because of the difference in the form of wing spot *vs.* courtship phenotypic scores, and was not pursued further.

### Traits for QTL analysis

As previously described (Yeh *et al.* 2006), approximately half of the backcross progeny males lacked wing spots. As a result, the backcross distributions of wing spot size measurements were skewed from the normal distribution. Normality of trait value distribution is a basic assumption of continuous variables in most QTL mapping methods. Because we could not transform the backcross progeny spot size distributions to normal distributions, we analyzed the wing spot trait in three ways, which we refer to as Spot Presence, Spot Size 1, and Spot Size 2. In Spot Presence, the wing spot trait was scored as presence (1) or absence (0), regardless of the size of the spot, and categorical IM was performed. In Spot Size 1, the full backcross spot size datasets were used, including individuals with no wing spot pigmentation. In Spot Size 2, only individuals with pigment in the wing spot area were used. Both IM and CIM were performed on the Spot Size 1 and Spot Size 2 backcross datasets.

For the Courtship Score trait, we used a numerical system with 0 referring to *D. gunungcola*−like courtship and 4 referring to *D. elegans*−like courtship. The interspecific difference in courtship behavior also can be dissected into several discrete elements. Therefore, we also scored the presence or absence of three courtship elements, Wing Display, Circling, and Body Shaking in backcross males and analyzed them using categorical IM. These courtship elements were described previously in detail (Yeh *et al.* 2006).

### Epistatic interaction analysis

Pairwise interactions between markers were tested using analysis of variance. We used three-way contingency table tests for Spot Presence, least square tests for Spot Size 1, and logistic regression for Courtship Score. Bonferroni corrections for multiple tests were applied by dividing *P* values by 820 (the number of interaction tests performed for each backcross dataset). Pairwise marker interaction tests were performed using R version 2.8.1 (http://www.r-project.org).

## Results

### Linkage map and karyotypes of *D. elegans/D. gunungcola*

Six linkage groups were identified in each backcross set (Figure S1 and Figure S2). The linkage groups in this map correspond to the six Muller/Sturtevant/Novitsky elements A-F (Powell 1997) with A corresponding to the X chromosome. Hereafter, linkage group A is referred to as X and the autosomes are referred to by the letters B through F. Many rearrangement differences from *D. melanogaster* are apparent (for example, *y* is near the tip of the X in *D. melanogaster* but in the center of the X in *D. elegans/D. gunungcola*). The two backcross analyses produced generally similar linkage maps except that the X was substantially longer in the *gunungcola* backcross (94.7cM) than in the *elegans* backcross (62.2 cM). This could be due to differential recovery of recombinant genotypes in the two backcrosses, possibly as a result of epistatic fitness interactions among loci. Although the length of our *D. elegans/D. gunungcola* maps are likely to be underestimated due to incomplete coverage of the chromosome ends, the total length estimates (*elegans* backcross 284.9 cM, *gunungcola* backcross 315.7 cM) are in the range of *D. melanogaster* (258.9 cM) and *D. mauritiana* (462.9 cM) (True *et al.* 1996).

The linkage map is consistent with the mitotic karyotype of *D. elegans* and *D. gunungcola*, which consists of four pairs of rods, one pair of dot-like chromosomes, and a pair of sex chromosomes (Figure S3) (Deng *et al.* 2007). All chromosomes in the *D. elegans/D. gunungcola* genome are thus inferred to be acrocentric, and their centromere-telomere orientations are unknown.

### At least three QTL underlie wing spot divergence

**Figure 1:**
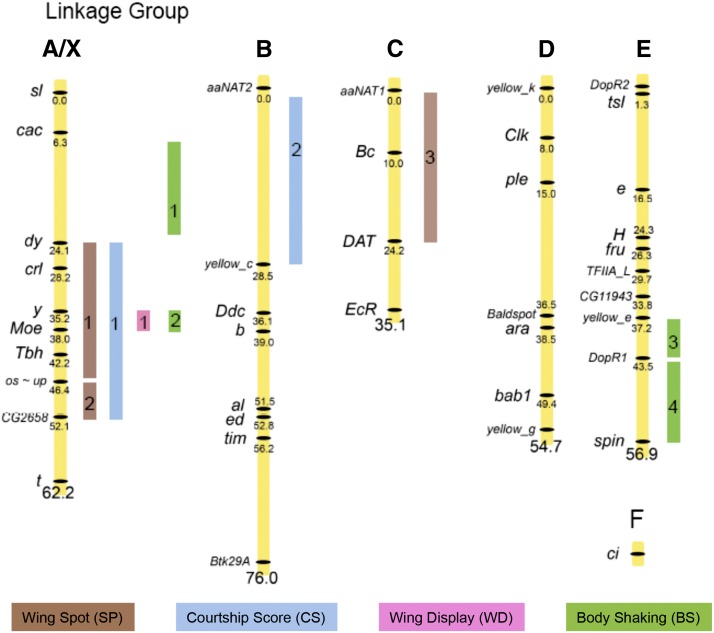
Summary of quantitative trait loci (QTL) results from *elegans* backcross males. Designations A through E correspond to Muller’s elements. Marker loci (on left of linkage groups) are named using the same names as the presumed orthologous *D. melanogaster* gene sequences used to develop the *D. elegans/D. gunungcola* markers. Intervals containing putative QTL are indicated on right of linkage groups (see also Table 2, Figure 3, and Figure 4). See Figure 2 for CIM maps of Spot Size 1, Spot Size 2, and Courtship Score datasets. See Figure 3 for IM maps of Wing Display, Circling, and Body Shaking datasets.QTL results from *gunungcola* backcross males are shown in Figure S4.

**Figure 2:**
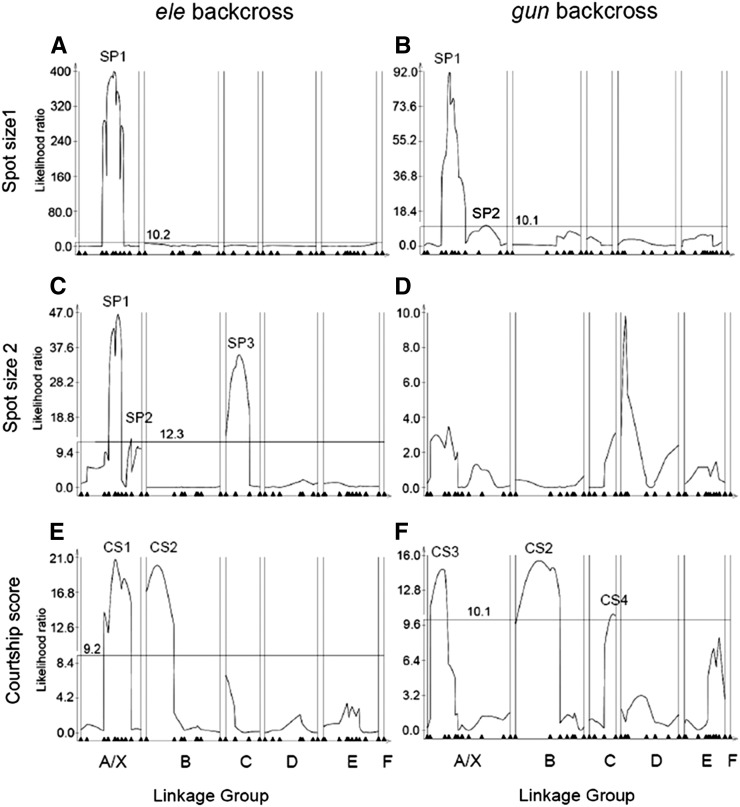
Composite interval maps for *elegans* (left) and *gunungcola* (right) backcross populations. A., B. Spot Size 1, C., D. Spot Size 2, E., F. Courtship Score. Horizontal lines in each plot indicate LR significance thresholds (see the section *Materials and Methods*). Marker positions are shown as black triangles at the bottom of each map.

The positions of all detected QTL mapped onto the *elegans* (*ele*) and *gunungcola* (*gun*) backcross linkage maps are shown in Figure 1 and Figure S4, respectively. The male wing spot backcross datasets were scored in three different ways: a binary score (Spot Presence), spot size across the full data set (Spot Size 1), and spot size only among males with wing spots (Spot Size 2). Both IM and CIM methods were used for Spot Size 1 and 2 and only IM was used for Spot Presence (see the *Materials and Methods*). Putative QTL with their positions and effects are listed in Table 2. Figure 2, A and B shows the CIM maps of the *ele* and *gun* backcrosses respectively for Spot Size 1. Figure 2, C and D shows the CIM maps for the *ele* and *gun* backcrosses, respectively, for Spot Size 2. The Spot Presence analysis (Table 2) gave similar results to Spot Size 1. Many of the QTL were detected in more than one analysis and/or in both backcrosses. In the right hand column of Table 2, we have given these QTL the same name to reflect the hypothesis that in these cases one QTL underlies the effect that appears in multiple analyses and/or in both backcrosses. IM maps for Spot Size 1, Spot Size 2, and Courtship Score are shown in Figure S5.

**Table 2:** List of detected QTL associated with divergence of male wing spots and courtship behavior between *D. elegans* and *D*. *gunungcola*

Trait	Backcross	Method	Chromosome	QTL Marker Interval*^a^*	QTL Interval Size, cM	QTL Peak*^b^*	LR	*R^2^**^c^*	Presumed QTL*^d^*
Spot Presence	*elegans*	IM	X	*sl-t*	62.2	37.0	24.5	NA	SP1
	*gunungcola*	IM	X	*sl-t*	94.7	30.0	52.0	NA	SP1
Spot Size 1*^e^*	*elegans*	IM	X	*sl-t*	62.2	36.2	379.6	0.947	SP1
		CIM	X	*dy-up*	22.3	36.2	399.8	0.924	SP1
	*gunungcola*	IM	X	*dy-os*	27.1	33.0	113.0	0.690	SP1
			X	*up-CG2658*	24.5	73.6	112.6	0.899	SP2
		CIM	X	*dy-os*	27.1	29.2	91.3	0.494	SP1
			X	*up-CG2658*	24.5	70.6	10.8	0.057	SP2
Spot Size 2*^f^*	*elegans*	IM	X	*crl-Tbh*	14.2	28.0	33.2	0.388	SP1
		CIM	X	*crl-Tbh*	14.2	38.0	46.6	0.384	SP1
			X	*up-CG2658*	5.7	51.9	13.2	0.140	SP2
			C	*aaNAT1-DAT*	24.2	13.5	35.6	0.287	SP3
	*gunungcola*	IM	None	−	−	−	−	−	
		CIM	None	−	−	−	−	−	
Courtship Score	*elegans*	IM	X	*cac-t*	55.9	37.1	21.3	0.140	CS1, CS3
			B	*aaNAT2-yellow c*	28.5	12.0	15.4	0.140	CS2
		CIM	X	*dy-CG2658*	28.0	35.7	20.8	0.113	CS1
			B	*aaNAT2-yellow c*	28.5	11.5	20.1	0.149	CS2
	*gunungcola*	IM	X	*cac-dy*	16.5	19.6	12.6	0.118	CS3
			B	*aaNAT2-Ddc*	51.0	28.0	12.4	0.155	CS2
			C	*aaNAT1-Bc*	6.0	3.0	10.0	0.097	CS4
			C	*Bc-DAT*	11.3	12.0	10.4	0.104	CS4
			C	*DAT-EcR*	12.9	23.3	10.9	0.115	CS4
		CIM	X	*cac-crl*	21.1	17.6	14.7	0.107	CS3
			B	*aaNAT2-Ddc*	51.0	27.0	15.5	0.134	CS2
			C	*Bc-DAT*	11.3	27.3	10.6	0.074	CS4
Wing Display	*elegans*	IM	X	*y-Moe*	2.8	38.0	11.0	NA	WD1
	*gunungcola*	IM	None	−	−	−	−	−	−
Circling	*elegans*	IM	None	−	−	−	−	−	−
	*gunungcola*	IM	X	*cac-dy*	16.5	9.0	10.7	NA	CI1
			C	*Bc-DAT*	11.3	13.0	11.1	NA	CI2
			C	*DAT-EcR*	12.9	20.0	11.0	NA	CI3
			D	*ple-bab1*	46.7	34.0	12.8	NA	CI4
			2 peaks			45.0	12.9	NA	CI4
Body Shaking	*elegans*	IM	X	*cac-dy*	17.8	23.0	12.5	NA	BS1
			X	*y-Moe*	2.8	38.0	13.0	NA	BS2
			E	*Yellow e-DopR1*	6.3	24.0	11.1	NA	BS3
			E	*DopR1-spin*	13.4	49.0	11.7	NA	BS4
	*gunungcola*	IM	None	−	−	−	−	−	−

**Figure 3:**
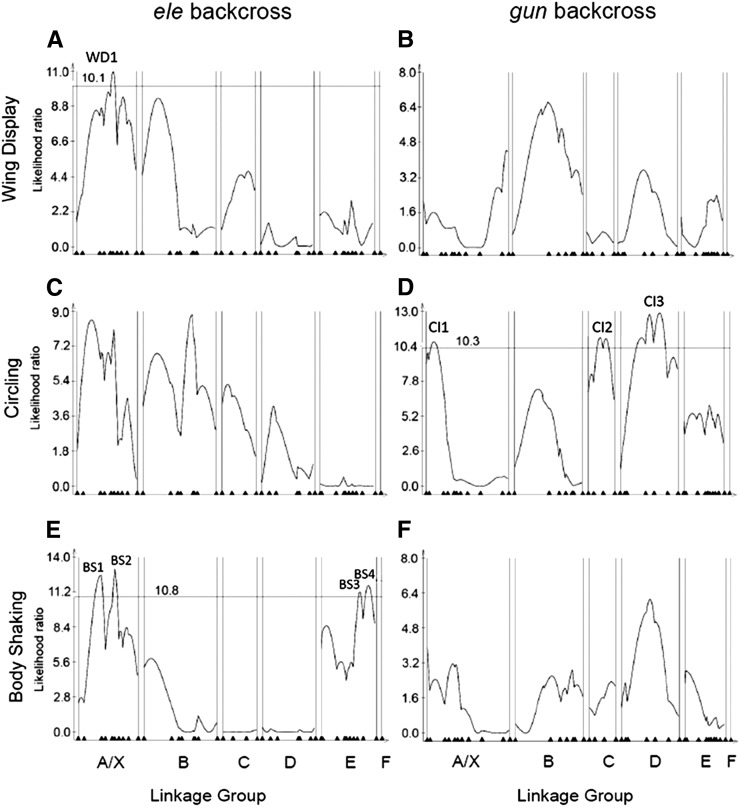
Interval mapping profiles for individual courtship elements in *elegans* (left) and *gunungcola* (right) backcross populations. A., B. Wing Display, C., D. Circling, E., F. Body Shaking. Horizontal lines in each plot indicate LR significance thresholds (see the section *Materials and Methods*). For B, C, and F, thresholds fell above all IM peaks. These thresholds were 9.00, 9.26, and 9.90, respectively.

CIM maps for Spot Size 1, Spot Size 2, and Courtship Score are shown in Figure 2. IM maps for Wing Display, Circling, and Body Shaking are shown in Figure 3. QTL, quantitative trait loci; LR, likelihood ratio;CIM, composite interval map; IM, interval map.

a QTL intervals given as linkage group (marker interval within linkage group). Linkage groups are named after the five conserved Muller elements, A-E with X corresponding to A. “Entire” indicates that the IM profile exceeded the significant threshold across the entire linkage group.

b QTL peak indicates the position in cM on the linkage map of the peak IM or CIM value in the interval corresponding to that QTL.

c *R^2^* represents the proportion of variance explained by the QTL, computed as R^2^ = (s^2^_0_ − s^2^_1_)/s^2^, where s^2^ = trait variance, s^2^_0_ = sample variance of residuals, s^2^_1_ = variance of residuals (Basten *et al.* 1999). *R^2^* is not applicable (NA) to binary data.

d See Figure 1, Figure 2, and Figure 3 for positions of QTL peaks.

e Spot set 1 consists of all individuals (see the *Materials and Methods*).

f Spot set 2 consists of only individuals with wing spots of any size (see the *Materials and Methods*).

All analyses support a major QTL for wing spots, SP1, on the X chromosome in the *y-Moe* interval. A QTL exceeding the significance threshold is found in this interval in all wing spot analyses except Spot Size 2 in the *gun* backcross (Figure 2D), which shows a subthreshold peak in this interval. On the right end of the X, there is also evidence of a peak in both backcrosses for Spot Size 2 but again the peak only reaches the threshold in the *ele* backcross. This QTL is also supported in the Spot Size 1 analysis in the *gun* backcross. We have designated this QTL as SP2. CIM mapping of Spot Size 2 in the *ele* backcross shows a QTL in the *aaNAT1-DAT* interval of linkage group C, SP3. Taken together these analyses indicate that at least three QTL determine the wing spot difference between *D. elegans* and *D. gunungcola*. The single autosomal QTL, SP3, appears only in the Spot Size 2 analysis, which excludes backcross males without wing spots (see the section *Materials and Methods*). This finding suggests that SP3 may be associated only with size (or intensity) of the wing spot whereas the X-linked QTL may be involved in determining both wing spot size and wing spot presence. The significance threshold for CIM of Spot Size 2 in the *gun* backcross (10.1 for both CIM; Figure 2 and IM analysis; Figure S5) was greater than all of the peaks. Thus our ability to detect QTL in that analysis was low, likely due to a small sample size of only 44 individuals in the *gun* backcross with measurable spot sizes.

The *R^2^* values in Table 2, which are computed for the traits scored as continuously variable, indicate the relative contributions of the detected QTL in terms of the percent variance accounted for by each QTL. For the wing spot analyses, SP1 consistently shows the largest effect (*R^2^* = 0.384−0.947) and in some cases is the only QTL detected (in some analyses SP1 and SP2 are not separable). *R^2^* values for SP2 ranged from 0.057 to 0.899. The only estimate of *R^2^* for SP3 was 0.287, suggesting a small-to-moderate effect. In the CIM analyses, these three QTL account for the majority of the variation in wing spot size. For example, for CIM of Spot Size 2 in the *ele* backcross, the three QTL account for 81.1% of the variation.

### At least four QTL underlie species difference in courtship score

CIM QTL maps of courtship score are shown in Figure 2, E and F for the *ele* and *gun* backcrosses, respectively, and positions and effects sizes of QTL for all analyses are listed in Table 2. IM maps of Courtship Score for the two backcrosses are shown in Figure 5SE and Figure 5SF. The two backcross datasets both provide support for a major effect of the X chromosome. In the *ele* backcross, the broad *dy-CG2658* interval is implicated, which we designate CS1. In the *gun* backcross, the more leftward *cac-crl* interval is implicated, which we designate CS3. The distinctly separate peaks of CS1 and CS3 are seen in both CIM (Figure 2, E and F) and IM maps (Figure 5SE and Figure 5SF), which suggests that the two backcross analyses are detecting distinct QTL. CIM and IM analyses of backcrosses support an additional QTL, CS2, in the left portion of linkage group B. The peaks corresponding to this QTL are substantially different between the *elegans* (28.5 cM) and *gunungcola* (51.0) backcrosses. This possibly indicates that two QTL are present on the left portion of linkage group B. However, given the large amount of overlap, we provisionally consider this evidence for a single QTL. Finally, the *gun* backcross analysis indicates a significant QTL peak on the right end of linkage group C, CS4. This is also reflected in the IM analysis of the *gun* backcrosses as a series of three adjacent peaks on this linkage group (Figure S5F). We provisionally interpret this as evidence for one QTL although multiple QTL may be present.

Unlike the wing spot analyses, the QTL detected in analysis of male courtship score generally had small and fairly uniform *R^2^* values. CS2 consistently exhibited the strongest effect in the CIM analyses (*R^2^* = 0.134-0.149). The effects of the other Courtship Score QTL in CIM were also fairly uniform and ranged from *R^2^* = 0.074-0.113. Overall, a large percentage of variance in Courtship Score was not accounted for in the CIM analyses (73.8% in the *ele* backcross and 68.5% in the *gun* backcross).

### Individual courtship elements show distinct QTL architectures

In addition to scoring overall courtship, we scored the backcross populations for the presence and absence of three male courtship elements which are exhibited by *D. elegans* but not by *D. gunungcola*: wing display, circling, and body shaking (see *Materials and Methods*). Because these traits were scored as binary or ordinal characters (see *Materials and Methods*) only IM analysis was used for QTL mapping. This method does not provide *R^2^* values for estimating absolute QTL effect but for analyses in which multiple QTL are detected, relative magnitudes of QTL can be discerned by comparing LR values (see Table 2). IM profiles for these traits are shown in Figure 3 and putative QTL positions are summarized in Table 2. For Wing Display (Figure 3, A and B), one QTL peak exceeded the significance threshold in the *ele* backcross and no peaks exceeded the threshold in the *gun* backcross. We denote this QTL in the *y-Moe* interval as WD1. WD1 may contribute to the effect of this interval on Courtship Score (CS1). Three QTL were detected for Circling behavior (Figure 3, C and D), all in the *gun* but not the *ele* backcross. CI1 is in the *cac-dy* interval of the X and thus may contribute to the Courtship Score QTL effect (CS3). CI2 is located medially on linkage group C in the *Bc-Ecr* region, and CI3 maps broadly between *ple* and *bab1* on linkage group D and is the only significant QTL detected on that linkage group. Both CI2 and CI3 have two prominent peaks of approximately equal height, suggesting the existence of multiple QTL in these intervals. Finally, for Body Shaking, we detected four QTL, all in the *ele* backcross (Figure 3, E and F). BS1 is in the *cac-dy* interval of the X chromosome. BS2 is in the *y-Moe* interval of the X and may contribute to the effect of CS1. BS3 is in the *yellow e-DopR1* interval on linkage group E and BS4 is in the *DopR1-spin* interval. Because these intervals are adjacent, they may reflect a single QTL, but because the likelihood ratio score falls substantially below the threshold between the peaks, we provisionally interpret this as evidence for two QTL.

### Evidence for epistatic genetic architecture in wing spots but not courtship score

**Figure 4:**
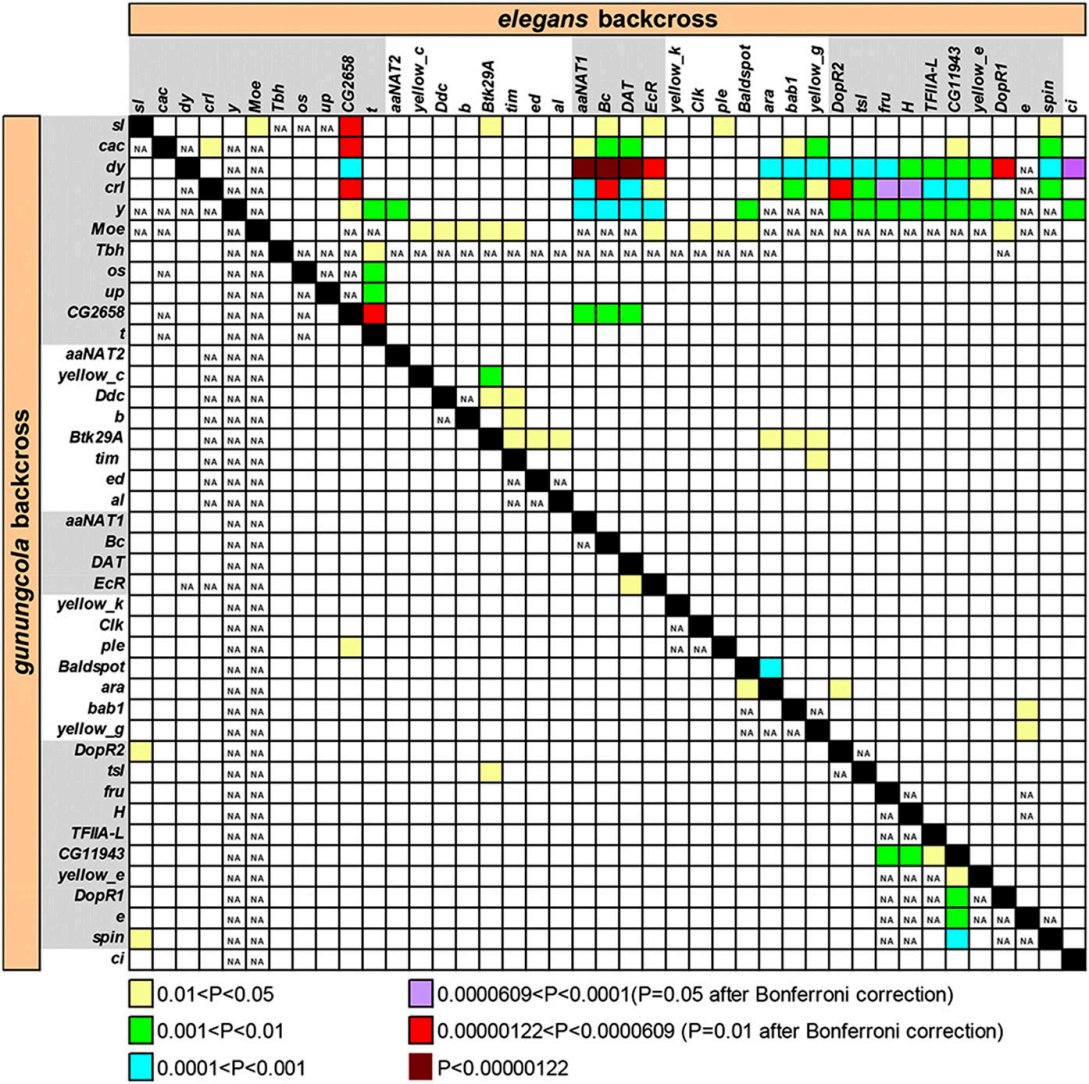
Pairwise marker interaction significance levels (see the section *Materials and Methods*) for Spot Size 1. *gunungcola* backcross results are in the lower left and *elegans* backcross results are in the upper right. NA indicates not enough data present for the analysis (nonzero wing spot size data points are needed, which are rare for some marker combinations in the *gun* backcross).

To examine whether epistasis among loci contributes to the genetic architecture of wing spot and courtship behavior divergence, we performed two-way analysis of variance for each pair of markers, analyzing the backcrosses separately. This was done for the Spot Presence, Spot Size 1, and Courtship Score datasets. Figure 4 shows the results for Spot Size 1. Several significant interactions were found after a highly conservative Bonferroni correction for the large number of tests (orange and red boxes in Figure 4). For this trait, pairwise marker interactions were only detected in the *ele* backcross. The failure to detect significant marker interactions in the *gun* backcross might be due to its smaller sample size or to weaker epistatic interactions in the gun genetic background. The *CG2658-t* interval of the X chromosome showed interactions with most of the rest of the X chromosome and the broad *sl-y* interval on the other end of the X chromosome (which may contain SP1) showed interactions with chromosome C (which contains SP3), chromosome F, and markers *DopR2* and *yellow e* on chromosome E. The clustering of significant interactions suggests that there is an underlying biological basis for these statistical interactions and that they are not due to chance. Interestingly, the *CG2658-t* region on X and the interacting markers on E do not correspond to any wing spot QTL detected using CIM or IM, which suggests the presence of additional QTL with primarily epistatic effects on wing spot divergence.

In contrast to the Spot Size 1 analysis, neither the Spot Presence analysis (Figure S6) nor the Courtship Score analysis (Figure S7) provided evidence for strong pairwise interactions among markers. Although some clusters of significant marker interactions were found, none of the individual interactions were significant after Bonferroni correction.

## Discussion

### Genetic architecture of divergence in wing spot and courtship behavior

We have found that the genetic architecture of coordinately evolving male wing spots and courtship displays in *D. elegans* and *D. gunungcola* reflects a polygenic basis for both pigmentation and behavioral components. At least three QTL underlie species divergence in wing pigmentation, and at least seven QTL underlie species divergence in male courtship. Relatively little evidence for epistasis among loci was found underlying these traits, although the Spot Size 1 analysis (Figure 4) provided evidence that some epistatic interactions may contribute to species divergence in wing spots. Another general hallmark of epistasis or dominance (intralocus epistasis) is the observation of QTL effects in one but not both backcrosses. One of the three wing spot QTL (SP3) was only detected in the *elegans* backcross, and three of the four QTL for courtship score (CS1, CS3, and CS4) were only found in one backcross (Figure 2). Perhaps consistent with this latter finding, all three courtship component trait analyses (Figure 3) uncovered QTL sets only in one backcross. This finding suggests that the genes that underlie these courtship elements are strongly influenced by genetic background and that the species-specific alleles have different degrees of dominance. In particular, all of the QTL detected for Circling were in the *gun* backcross (Figure 3, C and D) and all of the QTL detected for Body Shaking were in the *ele* backcross (Figure 3, E and F). Because it is likely that the common ancestor of *D. elegans* and *D. gunungcola* possessed both of these traits (Prud’homme *et al.* 2006), these QTL mapping results suggest that the *D. elegans* Circling QTL alleles act dominantly and that *D. gunungcola* evolved loss of Circling by means of novel recessive alleles. On the other hand, *D. gunungcola* appears to harbor dominant alleles responsible for the suppression of Body Shaking in backcross hybrids, consistent with evolution of gains of function at these loci in the *D. gunungcola* lineage. However, since these are male traits, QTL on the X chromosome that were only found in one backcross, but not in the other, cannot be explained by simple dominance. Instead, this result may imply a particular kind of epistatic interaction between X and autosomal factors. It is possible that some autosomal QTL or undetected factors act upstream of the effect of X chromosome QTL, which would then not be expressed in the absence of these autosomal factors.

Two potential caveats are involved with constructing the hybrid linkage maps used for QTL mapping in this study. First, as mentioned previously, it is likely that negative epistatic effects biased the recovery of genotypes in the backcross populations such that certain genotypes were not recovered or recovered less frequently than expected by chance. Second, to produce larger sample sizes for analysis, after determining that cytoplasmic background as a single genetic factor did not affect any of the trait means, we pooled across the two cytoplasmic backgrounds (grand-maternity of backcross progeny) to produce the two backcross sets that were analyzed. Both of these factors likely contributed to lower precision of the linkage maps. Negative epistasis among markers is likely in any linkage mapping effort based on an interspecies cross or distant outcross in which outbreeding depression is present, although these effects do not appear to be widely discussed in the linkage mapping literature. Recent studies of reciprocal crosses in wasps (Beukeboom *et al.* 2010) and mice (Dumont *et al.* 2011) suggest that recovery bias due to epistatic viability effects were negligible. QTL mapping from any cross can only reflect the recombinant genotypes that are recovered and the phenotypes with which they are statistically associated. The potential effects of cytoplasmic background on recombination have been indirectly studied in *Drosophila melanogaster*. Thoday and Boam (1956) found that decreases in X-linked recombination frequencies observed in inbred lines could be attributed to either cytoplasmic differences or X-linked chromosomal differences and were not due to inbreeding itself. Charlesworth and Charlesworth (1985a,b) studied a small but significant reciprocal cross effect on recombination frequency in a particular interval of the genome and found evidence for effects of both nuclear and cytoplasmic effects, with the cytoplasmic effects being likely attributable to *P-M* hybrid dysgenic effects. Recently, Dumont *et al.* (2011) found that linkage maps constructed from reciprocal crosses of mouse subspecies exhibited regional differences in recombination rate that could be attributed to either X-linked or mitochondrial factors. They estimated that approximately 19% of the genome showed significant map length differences between reciprocal crosses. Among the chromosomes showing significant map length differences, the average difference in map length between reciprocal crosses was 22%. On the other hand, the study of Beukeboom *et al.* (2010) found no significant differences between linkage maps generated from reciprocal interspecific crosses. Nevertheless, our study cannot rule out cytoplasmic (*i.e.*, mitochondrial) effects on local recombination rates.

Our results corroborate and extend our previous lower resolution study (Yeh *et al.* 2006) and complement findings on the molecular genetic basis of differences between *D. elegans* and *D. gunungcola* in male wing expression of *yellow* (Prud’homme *et al.* 2006; Yeh *et al.* 2006). Yellow protein is required for dark pigmentation in diverse *Drosophila* species (Sturtevant *et al.* 1929; Walter *et al.* 1991; Walter *et al.* 1996; Wittkopp *et al.* 2002b; Prud’homme *et al.* 2006). However, ectopic *yellow* expression alone is insufficient to produce novel pigment patterns (Wittkopp *et al.* 2002a). Consistent with this, we find that in addition to a major QTL in the genomic interval containing *yellow*, which must reflect at least in part the functional molecular differences mapped to *yellow* (Prud’homme *et al.* 2006), at least two other QTL contribute to interspecific wing spot divergence. We hypothesize that one or more of the genes underlying these QTL would be sufficient to cause novel pigmentation if expressed alone or coexpressed with *yellow*. Recently, Arnoult *et al.* (2013) reported that the transcriptional regulatory protein Distal-less (Dll) binds to an enhancer in the yellow gene that is required for Yellow protein expression in the spot. They also demonstrated that in *D. biarmipes*, expression of Dll in the spot region modulates the size and intensity of wing pigmentation through its binding of the yellow spot enhancer. Intriguingly, the SP3 QTL broadly maps to the genomic region (aaNAT1 to DAT; corresponding to cytological positions 53C7-8 to 60B2 on chromosome arm 2R) that is expected to contain the *D. elegans/D. gunungcola Dll* locus. Future fine-scale mapping should determine whether this QTL maps to *Dll*.

There has been a great deal of recent interest and research into the genetics of animal melanin pattern evolution (Majerus and Mundy 2003; True 2003; Wittkopp *et al.* 2003; Kelsh 2004; Mundy 2005; Wittkopp and Beldade 2009; Kronforst *et al.* 2012). In particular, evolutionary geneticists would like to determine whether independent evolution of similar melanin patterns (or losses of patterns) involves genetic changes at the same gene or genes or whether a large menu of possible genetic avenues are available for pigment pattern evolution. In mammals and birds, many cases of melanism are caused by amino acid substitutions in the Melanocortin 1 receptor, which controls the shift from pheomelanin to eumelanin production in melanocytes (Majerus and Mundy 2003), although cases in which Melanocortin 1 receptor is not implicated have been reported (Steiner *et al.* 2009).

In *Drosophila*, several genes in addition to *yellow* have been implicated in melanin pattern development, variation, and/or evolution. The transcription factors Engrailed (Gompel *et al.* 2005) and Dll (see above) have also been shown to play regulatory roles in Yellow expression in the wing spot. *ebony*, which encodes an enzyme that converts the melanin precursor dopamine to the precursor of yellowish, nonmelanized sclerotin, n-β-alanyl dopamine, has been found to underlie variation in body melanization within *D. melanogaster* (Pool and Aquadro 2007; Takahashi *et al.* 2007). *ebony* has also been shown to be expressed in the nonmelanized wing areas and down-regulated in wing spot regions in wing spot−bearing species (Wittkopp *et al.* 2002a). Regulatory evolution of *tan*, which encodes the enzyme catalyzing the reverse of the Ebony step (True *et al.* 2005), has been shown to underlie a major QTL contributing to abdominal pigmentation divergence between *D. yakuba* and *D. santomea* (Carbone *et al.* 2005; True *et al.* 2005; Jeong *et al.* 2008). Lastly, variation in male-specific abdominal pigmentation within *D. malerokotliana* is found due largely to three QTL that do not correspond positionally to any known *Drosophila* pigmentation genes (Ng *et al.* 2008). Our results are consistent with the previously known role of *yellow* but do not appear to implicate *ebony* or *tan* in wing spot divergence. It seems clear that multiple evolutionary genetic paths of gain and loss of pigmentation elements exist in *Drosophila*, and this may be true in other insects as well. Furthermore, since many of the QTL in these studies do not correspond to known melanin patterning genes, a number of important functional loci are likely yet to be discovered.

Species divergence in Courtship Score was found to be caused by at least seven QTL. However, most of the variance in Courtship Score (68.5–73.8%) was not accounted for in the CIM analyses. One possible explanation for this is that our courtship scoring system might not have effectively captured all of the variation among genotypes. Alternatively, there may be a large number of QTL of relatively minor effect that were not detectable with the scale and resolution of this study. Four of the seven courtship score QTL (CS1, CS3, CS6, and CS7) mapped closely to QTL for individual courtship components (see Figure 1 and Figure S4). These results are consistent with our earlier study (Yeh *et al.* 2006) and illustrate how courtship is itself a composite of genetically distinct sub-behaviors that may have evolved sequentially. Because Courtship Score was based on presence and absence of individual courtship elements, the QTL results for Courtship Score are not independent from those of the individual elements. A reasonable expectation then would be that the QTL for the individual elements should all lie within the set of QTL for courtship score. Although there are large overlaps, QTL on chromosome D for circling (Figure S4) and on E for Body Shaking (Figure 1) mapped to areas in which the Courtship Score QTL score did not exceed the significance threshold. This may have been due to low QTL detection power for Courtship Score. Sub threshold peaks for Courtship Score were seen on chromosomes D and E in the *gunungcola* backcross (Figure 2F), which might correspond to the Circling and Body Shaking QTL, respectively. Future analyses concentrating on individual courtship components should be fruitful in dissecting the molecular bases of each of the courtship components. A more quantitative treatment of individual courtship elements (*e.g.*, Cande *et al.* 2012) would also likely increase genetic resolution in future studies.

Various methods have been used to test whether apparent clustering of QTL for different traits is significant compared with results expected by chance alone (Jiang and Zeng 1995; Orr 1998; Kao *et al.* 1999; Hawthorne and Via 2001, Fishman *et al.* 2002; Hall *et al.* 2006). An alternative method to these for determining whether there is significant overlap between the QTL positions for two traits is to use a Fisher’s Exact Test with a 2×2 contingency table (Sokal and Rohlf 1995). In the *ele* backcross, 23 of 285 1-cM intervals (8.1%) exhibit QTL for both wing spot (including the results of CIM from Spot size 1 and Spot size 2) and courtship score (the results of CIM), which is significantly greater than expected (9 1-cM intervals based on the probability of overlap from 47 for wing spot and 52 for courtship; Fisher’s exact test, two-tailed *P* = 0.0168). In the *gun* backcross, 3 out of 316 1-cM intervals (0.9%) exhibit QTL for both wing spot and courtship score, which is not different from expected (9 1-cM intervals based on the probability of overlap from 47 for wing spot and 52 for courtship; Fisher’s exact test, two-tailed *P* = 0.1422). Therefore, we conclude that the QTL for wing spots and courtship score are clustered in the *ele* backcross, but not in the *gun* backcross.

In the case of the QTL cluster in the *yellow* region on the X chromosome, containing the QTL BS2, SP1, CS1, and WD1, the *yellow* gene itself is a candidate for involvement in male courtship behavior divergence between species. If *yellow* has pleiotropic effects on both male pigmentation and courtship it could greatly facilitate the coordinated evolution of these traits that has occurred in *Drosophila*. However, due to limits in genetic resolution, QTL studies tend to give an overestimated view of pleiotropy (Wagner and Zhang 2011). An alternative way to investigate possible pleiotropic effects is to examine mutational phenotypes. In *D. melanogaster*, *yellow* mutants exhibit well known deficiencies in male mating success that appear to be due to a defect in wing extension during courtship (Bastock 1956; Burnet *et al.* 1973; Drapeau *et al.* 2003). Recent studies by Drapeau *et al.* (2003, 2006) have shown that sexually dimorphic expression of Yellow in a small subset of brain cells is associated with the wing extension defect in *yellow* males and that both the expression and phenotype can be rescued by Supplemental expression of *yellow*. The brain expression of *yellow* is downstream of the sex determination transcription factor Fruitless and transcriptional activation of the *yellow* pattern occurs through a 300-bp *cis*-regulatory element upstream of the *yellow* promoter (Drapeau *et al.* 2006). However, males from *D. elegans yellow* mutant strains that we have isolated do not appear to have lower mating success compared to wild type males (S.-D. Yeh, E. Hill-Burns, and J. R. True, unpublished data). We have not yet examined these lines for differences in wing movement during courtship. Fine scale mapping of the X-linked QTL in *D. elegans/D. gunungcola* is needed to determine whether divergence of *yellow* or other genes underlies evolutionary genetic correlations between male pigmentation and courtship behavior. An alternative possibility to pleiotropy is that a tight cluster of two or more genes affects both pigmentation and behavior and may promote their coordinated evolution. Such ‘supergenes’ have been found to control major aspects of mimetic color patterns in *Heliconius* butterflies (Joron *et al.* 2006, 2011; *Jones et al.* 2012).

### Genetic scenarios of wing spot loss in the *elegans* species subgroup

**Figure 5:**
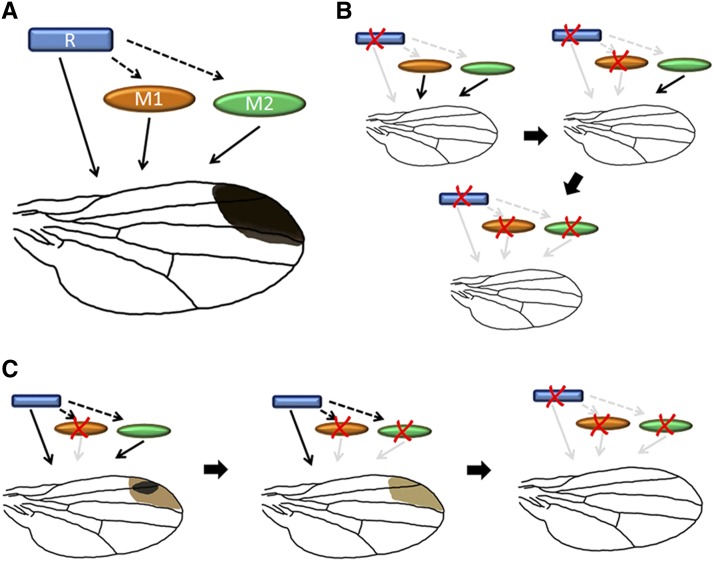
Possible genetic scenarios of wing spot loss in *D. gunungcola*. (A) Genetic factors influencing the development of the wing spot are hypothesized to consist of one or more “Regulator” factors (blue rectangle) that determine the area of pigment deposition by acting upon one or more “Modifier” factors (orange and green ellipsoids) that determine the darkness and size of the wing spot. (B) In the “Regulators-first” scenario, loss of the wing spot occurs through one or a small number of changes in regulator expression in the spot area and subsequent loss of modifier expression occurs due to the relaxation of selection or genetic drift. (C) In the “Modifiers-first” scenario, the trajectory of wing spot loss is more gradual due to accumulation of sequential changes in both “Regulator” and downstream “Modulator” genes such as pigmentation enzymes.

Our results suggest that for wing spots, epistatic interactions may be involved in determining the effects of divergent QTL loci on the phenotype. Two different types of gene functions could be envisioned as part of such a mechanism. One type of locus, a “regulator,” would determine whether pigment could be deposited in the wing spot area, the other type, a “modifier,” would control the intensity (including the darkness and size) of pigmentation in the wing spot area (Figure 5A). Given such a functional division, the loss of wing spots in the *D. gunungcola* lineage could occur by two different scenarios: “regulators first” or “modifiers first.” In the “regulators first” scenario, changes of upstream regulators might occur first to knock out the expression of melanin pathway genes in the wing spot area. Mutations in wing-spot-specific regulatory regions of modifiers would subsequently accumulate over time, due to the relaxation of selective pressure or genetic drift (Figure 5B). Alternatively, in the “modifiers first” scenario, loss of modifier gene expression in the wing spot region might have occurred and been fixed in the population before the changes of regulators (Figure 5C). In the former scenario, the pigmentation in wing spot area would disappear before the fixation of any changes in downstream pigmentation genes. In the later scenario, the pigmentation intensity might have been lost gradually with or without later changes in regulator genes.

Information on the function of wing pigmentation during courtship would be relevant to ascertaining which of these scenarios occurred during the divergence of these two species. Wing spots in the Oriental *melanogaster* species group appear to be a sexually-selected trait maintained by female preference (Fuyama 1977, 1979; Kopp and True 2002; Hegde *et al.* 2005). Female preferences on wing spots are expected to have influenced the evolution of this trait. Assuming the common ancestor of *D. elegans* and *D. gunungcola* had a female preference for *D. elegans*−sized male wing spots, the nature of the evolutionary trajectory by which spots were lost would involve an interplay between female preference and the developmental genetic changes in the wing spot pathway modeled above. For example, if the loss of wing spots in *D. gunungcola* resulted from strong selection (sexual or natural; *i.e.*, males with no spots had the highest fitness), changes of “regulators” resulting in complete spot loss might evolve before changes in “modifiers.” On the other hand, if wing spots instead became a neutral trait in *D. gunungcola*, due to the loss of female preference for them or the loss of the wing display behavior, then changes in regulators and modifiers might be equally likely. These scenarios could be tested, once underlying loci are identified, by examining genetic variation at these loci in *D. gunungcola*. In the former scenario, we would expect to find that modifier genes would show little or no evidence for selection, at least in sequences important for wing spot expression, but that the regulatory gene(s) would show evidence of strong selection. Further elucidating the genetic changes underlying the loss of male wing pigmentation and courtship behavior in *D. gunungcola* will greatly help to distinguish among these possible trajectories.

## Supplementary Material

Supporting Information
