# Supplementary material for: The Genetic Architecture of Coordinately Evolving Male Wing Pigmentation and Courtship Behavior in Drosophila elegans and Drosophila gunungcola
Source: G3 (Bethesda). 2014 Aug 27;4(11):2079–93. doi: 10.1534/g3.114.013037 (PMC4232533; doi:10.1534/g3.114.013037)
Supplement: Supporting Information [file supp_4_11_2079__index.html]

The Genetic Architecture of Coordinately Evolving Male Wing Pigmentation and Courtship Behavior in Drosophila elegans and Drosophila gunungcola — Supporting Information 

# The Genetic Architecture of Coordinately Evolving Male Wing Pigmentation and Courtship Behavior in *Drosophila elegans* and *Drosophila gunungcola*

## Supporting Information for Yeh and True, 2014

**Files in this Data Supplement:**

- Supporting Information - Figures S1-S7, Table S1, and File S1 (PDF, 557 KB)
- Figure S1 - The genetic linkage map obtained from the *ele* backcross data set. (PDF, 423 KB)
- Figure S2 - The genetic linkage map obtained from the *gun* backcross set. (PDF, 423 KB)
- Figure S3 - Typical mitotic chromosome spreads of *D. elegans* and *D. gunungcola*. (PDF, 214 KB)
- Figure S4 - Summary of QTL results from *gunungcola* backcross males. (PDF, 466 KB)
- Figure S5 - Interval maps (IM) for *elegans* (left) and *gunungcola* (right) backcross populations. (PDF, 513 KB)
- Figure S6 - Pairwise marker interaction significance levels (see Materials and Methods) for Spot Presence. (PDF, 131 KB)
- Figure S7 - Pairwise marker interaction significance levels (see Materials and Methods) for Courtship Score. (PDF, 131 KB)
- Table S1 - List of the accession numbers of DNA sequences in this study. (PDF, 140 KB)
- File S1 - Raw data. (.xls, 157 KB)
