# Supplementary material for: The Genetic Architecture of Coordinately Evolving Male Wing Pigmentation and Courtship Behavior in Drosophila elegans and Drosophila gunungcola
Source: G3 (Bethesda). 2014 Aug 27;4(11):2079–93. doi: 10.1534/g3.114.013037 (PMC4232533; doi:10.1534/g3.114.013037)
Supplement: Supporting Information [file supp_g3.114.013037_013037SI.pdf]

G3·Genes | Genomes | Genetics

The genetic architecture of coordinately evolving male wing pigmentation and courtship behavior in *Drosophila elegans* and *D. gunungcola*

Shu-Dan Yeh and John R. True

Department of Ecology and Evolution, Stony Brook University, Stony Brook, NY 11794-5245, USA

Corresponding Author: John R. True Department of Ecology and Evolution, Stony Brook University, Stony Brook, NY 11794-5245, USA
e-mail: jrtrue@life.bio.sunysb.edu, phone: 631-379-7266

DOI: 10.1534/g3.114.013037

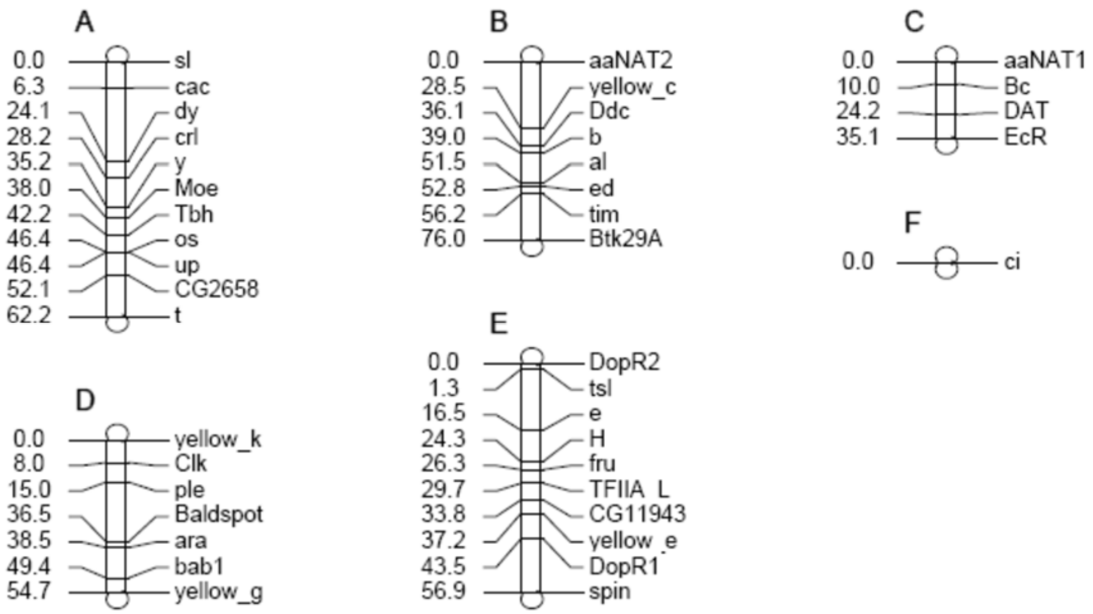

Figure S1 The genetic linkage map obtained from the *ele* backcross data set. Designations A through E correspond to Muller's elements.

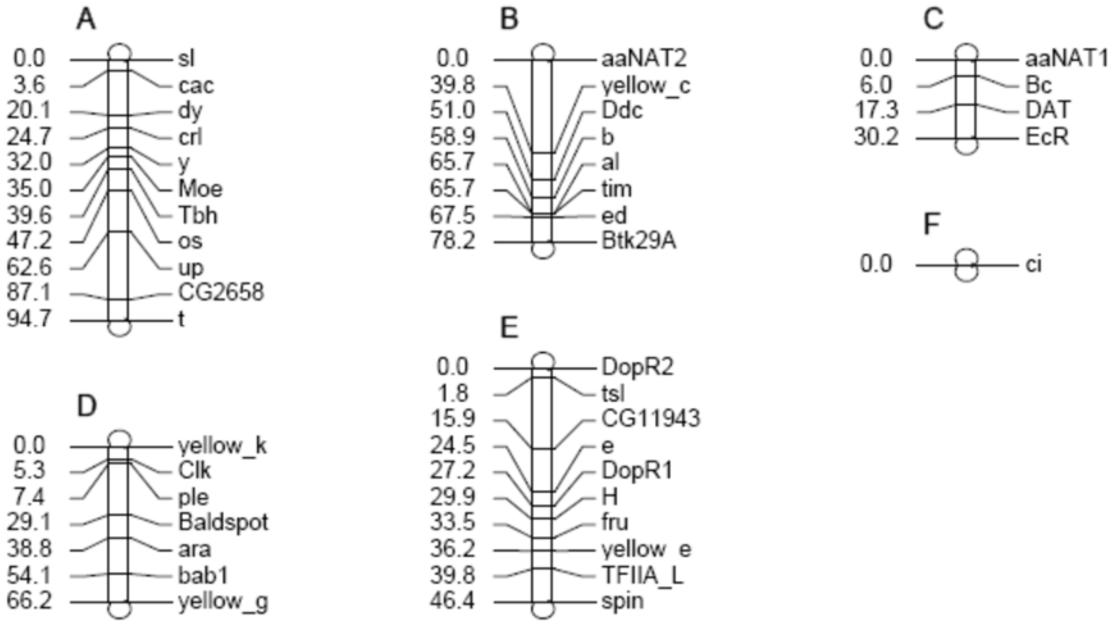

Figure S2 The genetic linkage map obtained from the *gun* backcross set. Designations A through E correspond to Muller's elements.

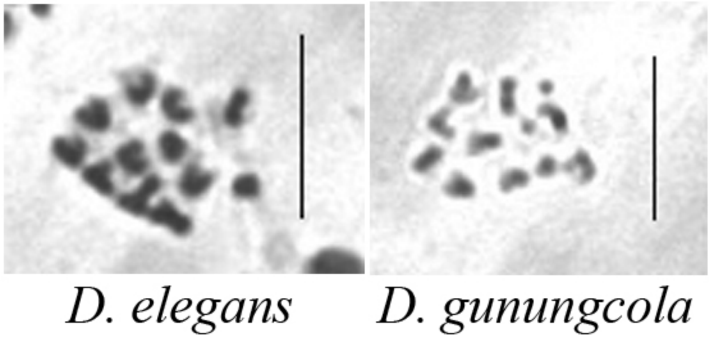

Figure S3 Typical mitotic chromosome spreads of *D. elegans* and *D. gunungcola.* In the spreads above, clusters of 12 elements are clearly apparent. It is difficult to distinguish the sex chromosome pair from the four rod pairs. In the *D. gunungcola* spread, the dot chromosomes (Muller/Sturtevant/Novitsky element F) can be seen in the upper right of the cluster. Scale bars: 20 microns. For a more detailed analysis of the karyotype of *D. elegans* and related species, see Deng et al. 2007.

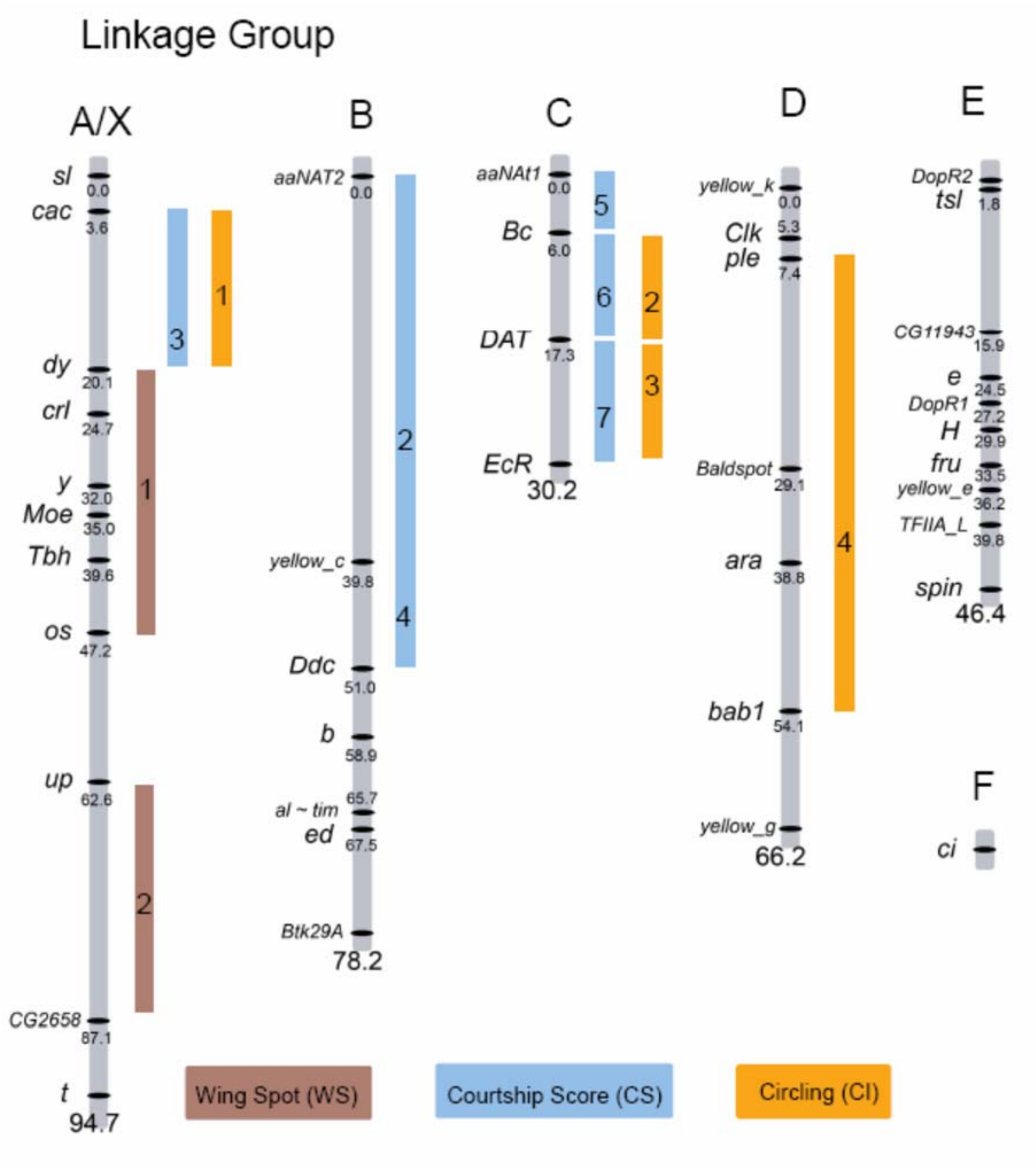

Figure S4 Summary of QTL results from *gunungcola* backcross males. Designations A through E correspond to Muller's elements. Marker loci (on left of linkage groups) are named using the same names as the presumed orthologous *D. melanogaster* gene sequences used to develop the *D. elegans/D. gunungcola* markers. Intervals containing putative QTL are indicated on right of linkage groups (see also Table 2 and Figs. 3, 4). See Figure 2 for CIM maps of Spot Size 1, Spot Size 2, and Courtship Score datasets. See Figure 3 for IM maps of Wing Display, Circling, and Body Shaking datasets.

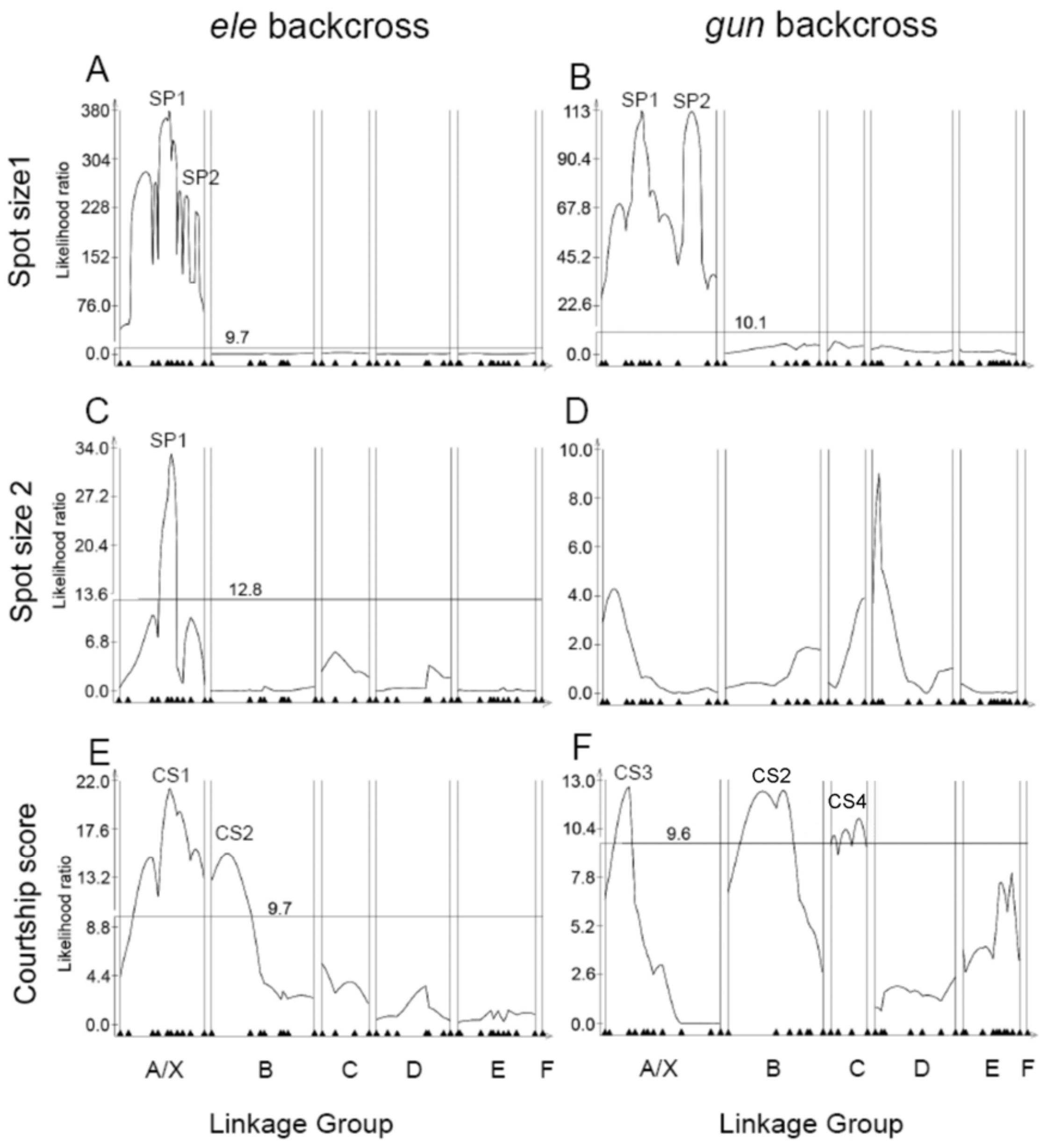

Figure S5 Interval maps (IM) for *elegans* (left) and *gunungcola* (right) backcross populations. A., B. Spot Size 1, C., D. Spot Size 2, E., F. Courtship Score. Horizontal lines in each plot indicate LR significance thresholds (see Materials and Methods).

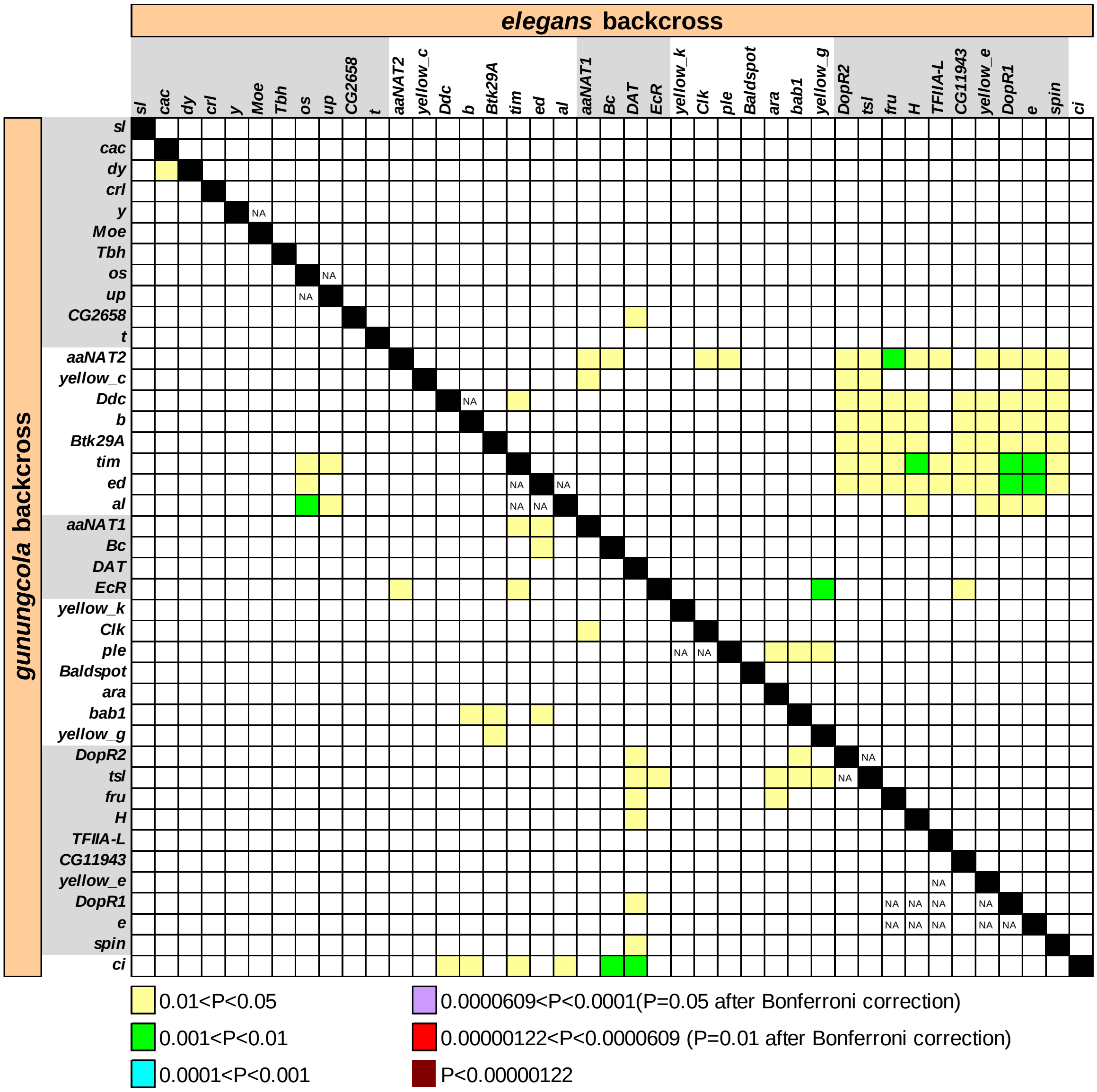

Figure S6 Pairwise marker interaction significance levels (see Materials and Methods) for Spot Presence. *gunungcola* backcross results are in the lower left and *elegans* backcross results are in the upper right.

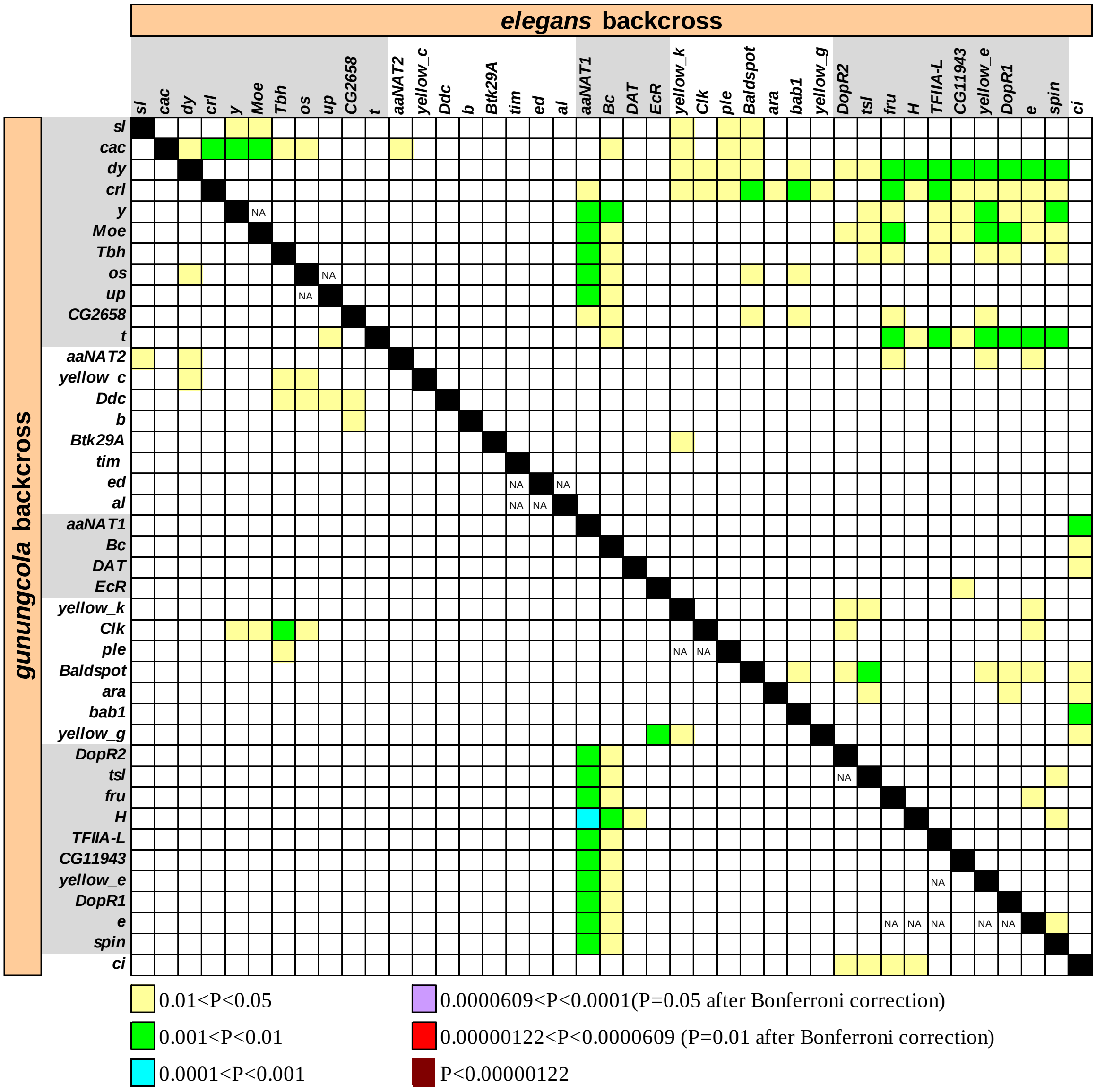

Figure S7 Pairwise marker interaction significance levels (see Materials and Methods) for Courtship Score. *gunungcola* backcross results are in the lower left half and *elegans* backcross results are in the upper right.

File S1

Raw Data

Available for download as an Excel file at http://www.g3journal.org/lookup/suppl/doi:10.1534/g3.114.013037/-/DC1

Table S1 List of the accession numbers of DNA sequences in this study.

Locus[#]	Species	GenBank Accession number
X		
yellow	*D. elegans*	FJ889358
	D. gunungcola	FJ889359
CG2658	*D. elegans*	FJ889360
	D. gunungcola	FJ889361
Tyramine beta hydroxylase	*D. elegans*	FJ889362
	D. gunungcola	FJ889363
Moesin	*D. elegans*	FJ889364
	D. gunungcola	FJ889365
tan	*D. elegans*	FJ889366
	D. gunungcola	FJ889367
*dusky(L)**	*D. elegans*	FJ889368
	D. gunungcola	FJ889369
*dusky(R)**	*D. elegans*	FJ889370
	D. gunungcola	FJ889371
cacophony	*D. elegans*	FJ889372
	D. gunungcola	FJ889373
upheld	*D. elegans*	FJ889374
	D. gunungcola	FJ889375
small wing	*D. elegans*	FJ889376
	D. gunungcola	FJ889377
courtless	*D. elegans*	FJ889378
	D. gunungcola	FJ889379
outstretched	*D. elegans*	FJ889380
	D. gunungcola	FJ889381
CG11943	*D. elegans*	FJ889382
	D. gunungcola	FJ889383
2L		
aristaless	*D. elegans*	FJ889384
	D. gunungcola	FJ889385
timeless	*D. elegans*	FJ889386
	D. gunungcola	FJ889387
echinoid	*D. elegans*	FJ889388
	D. gunungcola	FJ889389

Table S1 (Continued)

Locus	Species	Accession number
arylalkylamine N-acetyltransferase 2	*D. elegans*	FJ889390
	D. gunungcola	FJ889391
Btk family kinase at 29A	*D. elegans*	FJ889392
	D. gunungcola	FJ889393
black	*D. elegans*	FJ889394
	D. gunungcola	FJ889395
yellow c	*D. elegans*	FJ889396
	D. gunungcola	FJ889397
Dopa decarbosylase	*D. elegans*	FJ889398
	D. gunungcola	FJ889399
2R		
Ecdysone Receptor	*D. elegans*	FJ889400
	D. gunungcola	FJ889401
spinster	*D. elegans*	FJ889402
	D. gunungcola	FJ889403
Dopamine Transporter	*D. elegans*	FJ889404
	D. gunungcola	FJ889405
Black cells	*D. elegans*	FJ889406
	D. gunungcola	FJ889407
Dopamine N acetyltransferase	*D. elegans*	FJ889408
	D. gunungcola	FJ889409
3L		
bric a brac 1	*D. elegans*	FJ889410
	D. gunungcola	FJ889411
yellow g	*D. elegans*	FJ889412
	D. gunungcola	FJ889413
pale	*D. elegans*	FJ889414
	D. gunungcola	FJ889415
Clock	*D. elegans*	FJ889416
	D. gunungcola	FJ889417
aracaun	*D. elegans*	FJ889418
	D. gunungcola	FJ889419
yellow k	*D. elegans*	FJ889420
	D. gunungcola	FJ889421

Table S1 (Continued)

Locus	Species	Accession number
Baldspot	*D. elegans*	FJ889422
	D. gunungcola	FJ889423
3R		
yellow e	*D. elegans*	FJ889424
	D. gunungcola	FJ889425
Dopamine Receptor (1)	*D. elegans*	FJ889426
	D. gunungcola	FJ889427
fruitless	*D. elegans*	FJ889428
	D. gunungcola	FJ889429
Hairless	*D. elegans*	FJ889430
	D. gunungcola	FJ889431
ebony	*D. elegans*	FJ889432
	D. gunungcola	FJ889433
torso-like	*D. elegans*	FJ889434
	D. gunungcola	FJ889435
TfIIA-L	*D. elegans*	FJ889436
	D. gunungcola	FJ889437
Dopamine Receptor2	*D. elegans*	FJ889438
	D. gunungcola	FJ889439
4		
cubitus interruptus	*D. elegans*	FJ889440
	D. gunungcola	FJ889441

[#] The chromosome arm where loci reside in *D. melanogaster*.

*DNA sequence information between left and right primers of *dusky* is incomplete. Thus, the sequences obtained for designing the primer set of *dusky* are submitted separately.
