# Supplementary material for: The Genetic Architecture of Coordinately Evolving Male Wing Pigmentation and Courtship Behavior in Drosophila elegans and Drosophila gunungcola
Source: G3 (Bethesda). 2014 Aug 27;4(11):2079–93. doi: 10.1534/g3.114.013037 (PMC4232533; doi:10.1534/g3.114.013037)
Supplement: Supporting Information [file supp_g3.114.013037_FigureS3.pdf]

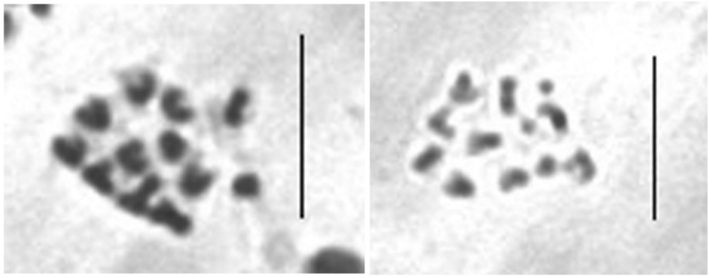

Figure S3 Typical mitotic chromosome spreads of *D. elegans* and *D. gunungcola.* In the spreads above, clusters of 12 elements are clearly apparent. It is difficult to distinguish the sex chromosome pair from the four rod pairs. In the *D. gunungcola* spread, the dot chromosomes (Muller/Sturtevant/Novitsky element F) can be seen in the upper right of the cluster. Scale bars: 20 microns. For a more detailed analysis of the karyotype of *D. elegans* and related species, see Deng et al. 2007.
