# Supplementary material for: The Genetic Architecture of Coordinately Evolving Male Wing Pigmentation and Courtship Behavior in Drosophila elegans and Drosophila gunungcola
Source: G3 (Bethesda). 2014 Aug 27;4(11):2079–93. doi: 10.1534/g3.114.013037 (PMC4232533; doi:10.1534/g3.114.013037)
Supplement: Supporting Information [file supp_g3.114.013037_FigureS4.pdf]

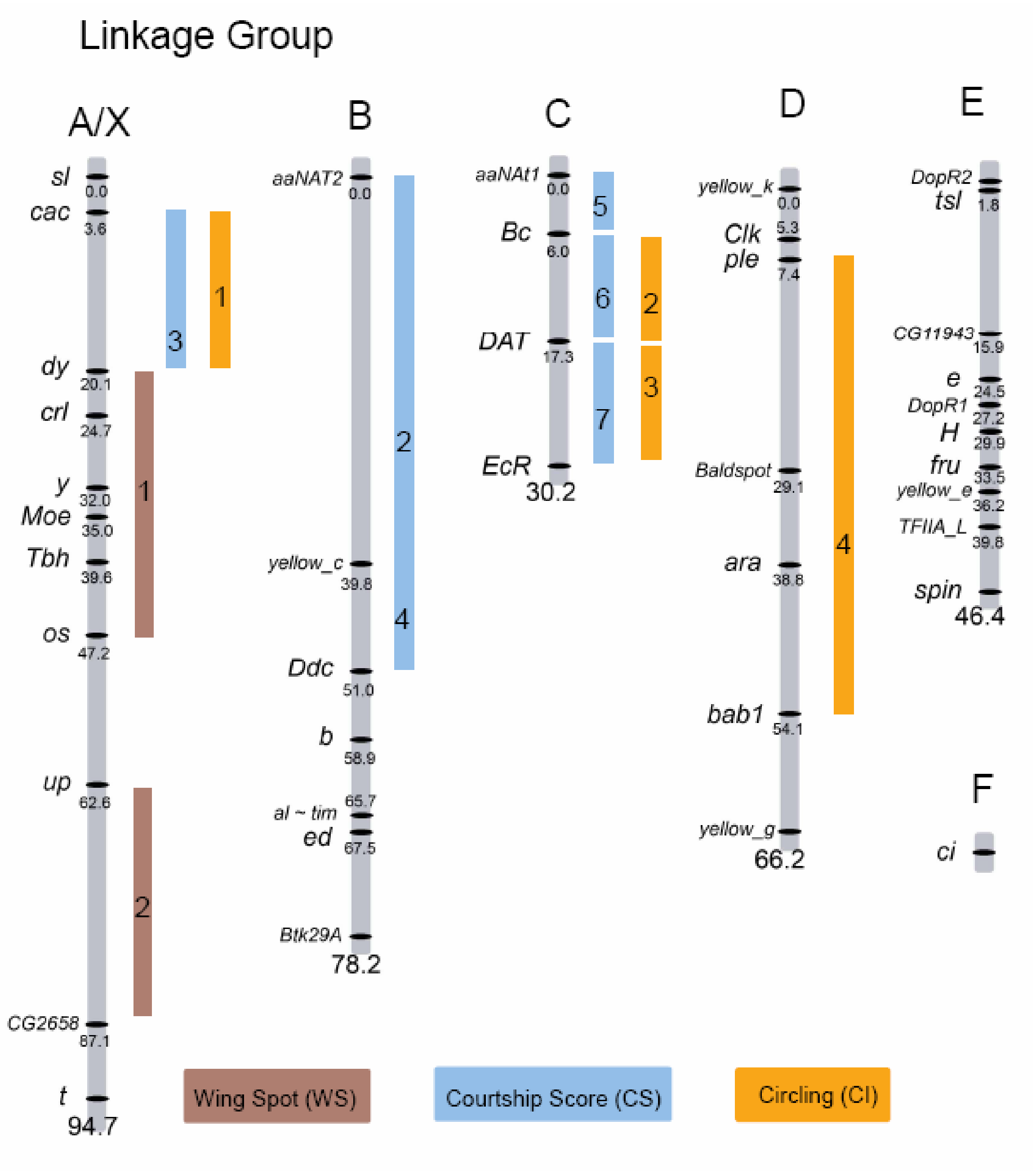

Figure S4 Summary of QTL results from *gunungcola* backcross males. Designations A through E correspond to Muller's elements. Marker loci (on left of linkage groups) are named using the same names as the presumed orthologous *D. melanogaster* gene sequences used to develop the *D. elegans/D. gunungcola* markers. Intervals containing putative QTL are indicated on right of linkage groups (see also Table 2 and Figs. 3, 4). See Figure 2 for CIM maps of Spot Size 1, Spot Size 2, and Courtship Score datasets. See Figure 3 for IM maps of Wing Display, Circling, and Body Shaking datasets.
