# Supplementary material for: The Genetic Architecture of Coordinately Evolving Male Wing Pigmentation and Courtship Behavior in Drosophila elegans and Drosophila gunungcola
Source: G3 (Bethesda). 2014 Aug 27;4(11):2079–93. doi: 10.1534/g3.114.013037 (PMC4232533; doi:10.1534/g3.114.013037)
Supplement: Supporting Information [file supp_g3.114.013037_FigureS5.pdf]

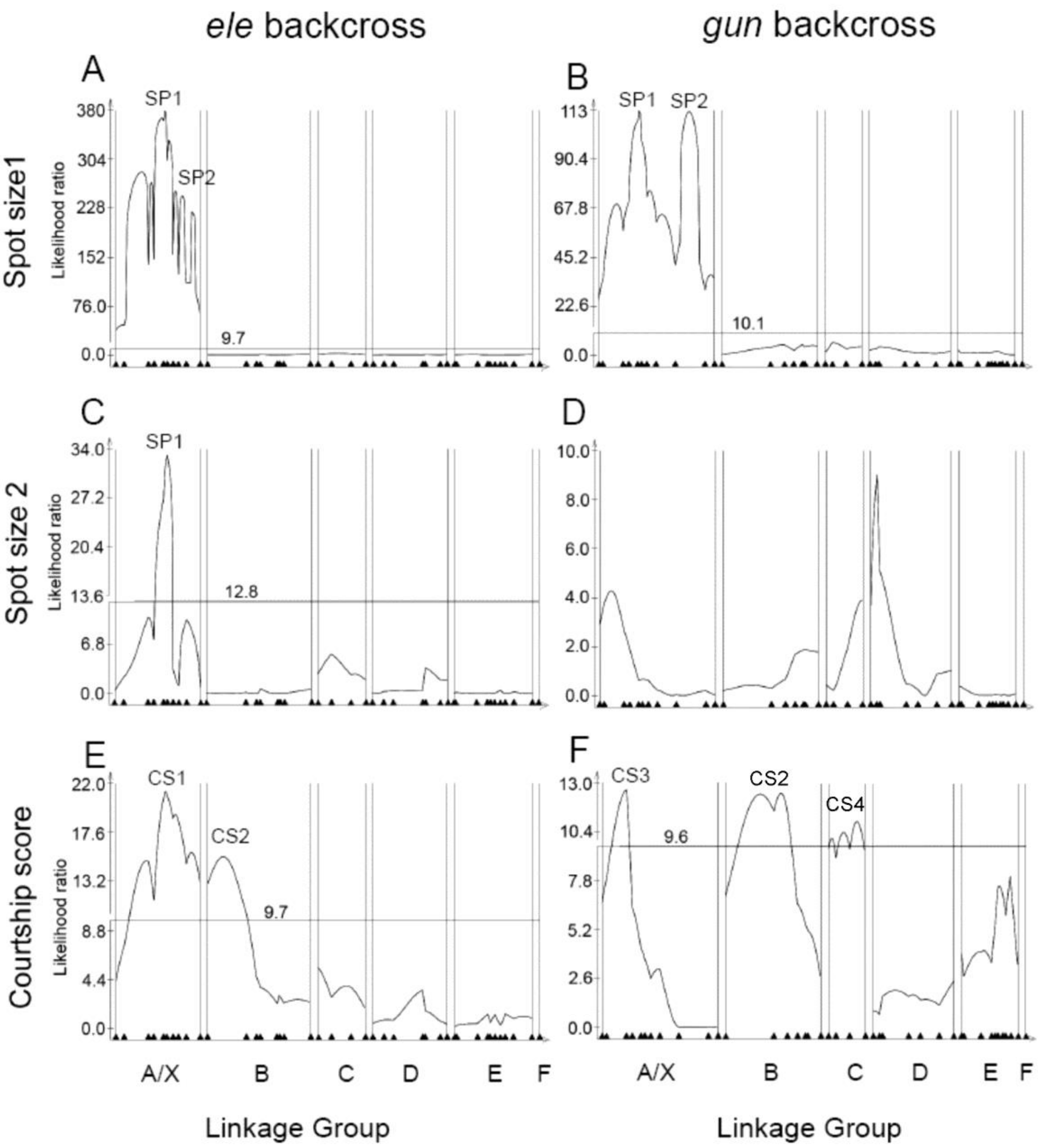

Figure S5 Interval maps (IM) for *elegans* (left) and *gunungcola* (right) backcross populations. A., B. Spot Size 1, C., D. Spot Size 2, E., F. Courtship Score. Horizontal lines in each plot indicate LR significance thresholds (see Materials and Methods).
