# Supplementary material for: The Genetic Architecture of Coordinately Evolving Male Wing Pigmentation and Courtship Behavior in Drosophila elegans and Drosophila gunungcola
Source: G3 (Bethesda). 2014 Aug 27;4(11):2079–93. doi: 10.1534/g3.114.013037 (PMC4232533; doi:10.1534/g3.114.013037)
Supplement: Supporting Information [file supp_g3.114.013037_FigureS7.pdf]

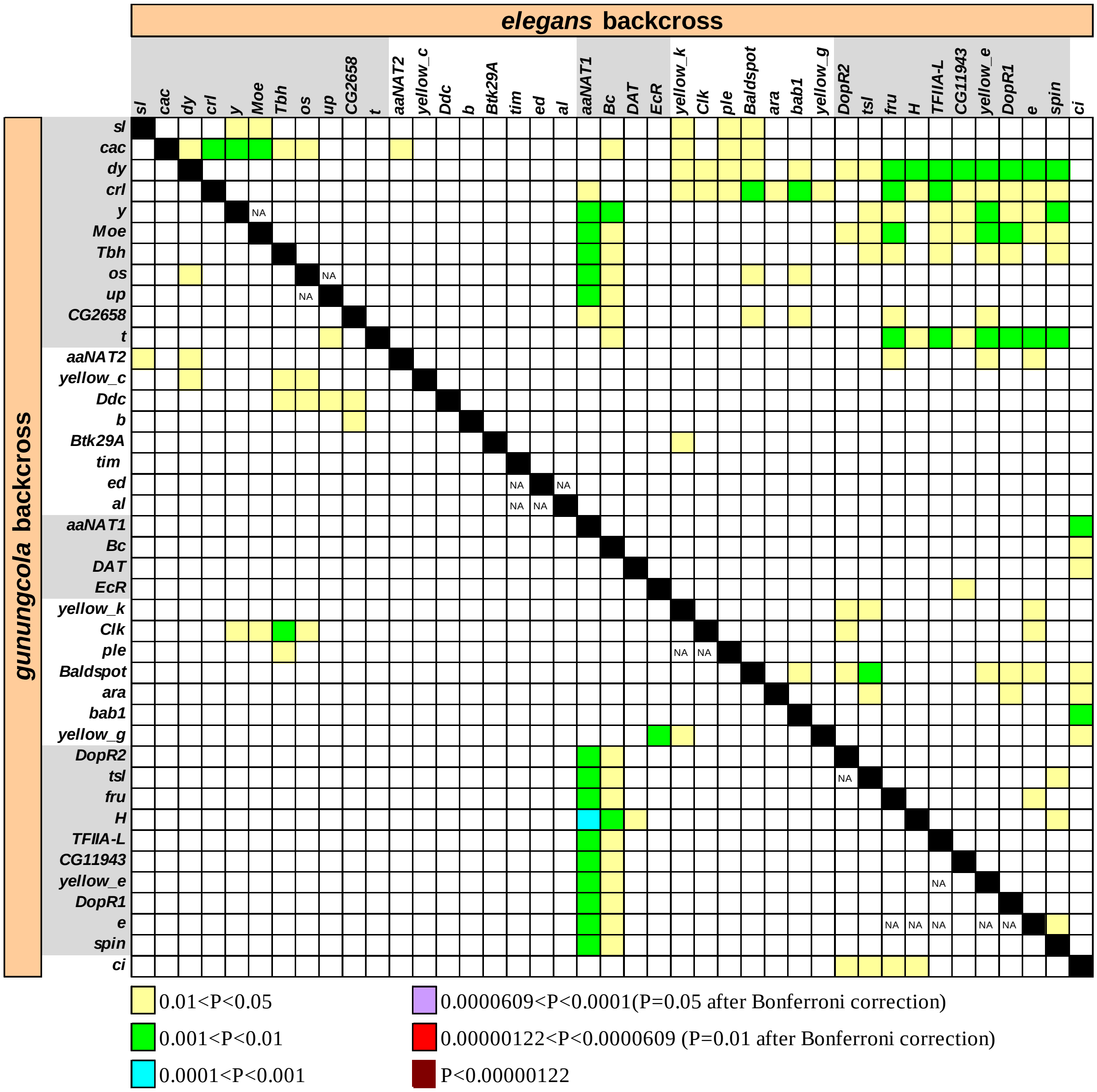

Figure S7 Pairwise marker interaction significance levels (see Materials and Methods) for Courtship Score. *gunungcola* backcross results are in the lower left half and *elegans* backcross results are in the upper right.
