# Supplementary material for: The Genetic Architecture of Coordinately Evolving Male Wing Pigmentation and Courtship Behavior in Drosophila elegans and Drosophila gunungcola
Source: G3 (Bethesda). 2014 Aug 27;4(11):2079–93. doi: 10.1534/g3.114.013037 (PMC4232533; doi:10.1534/g3.114.013037)
Supplement: Supporting Information [file supp_g3.114.013037_TableS1.pdf]

Table S1 List of the accession numbers of DNA sequences in this study.

Locus[#]	Species	GenBank Accession number
X		
yellow	*D. elegans*	FJ889358
	D. gunungcola	FJ889359
CG2658	*D. elegans*	FJ889360
	D. gunungcola	FJ889361
Tyramine beta hydroxylase	*D. elegans*	FJ889362
	D. gunungcola	FJ889363
Moesin	*D. elegans*	FJ889364
	D. gunungcola	FJ889365
tan	*D. elegans*	FJ889366
	D. gunungcola	FJ889367
*dusky(L)**	*D. elegans*	FJ889368
	D. gunungcola	FJ889369
*dusky(R)**	*D. elegans*	FJ889370
	D. gunungcola	FJ889371
cacophony	*D. elegans*	FJ889372
	D. gunungcola	FJ889373
upheld	*D. elegans*	FJ889374
	D. gunungcola	FJ889375
small wing	*D. elegans*	FJ889376
	D. gunungcola	FJ889377
courtless	*D. elegans*	FJ889378
	D. gunungcola	FJ889379
outstretched	*D. elegans*	FJ889380
	D. gunungcola	FJ889381
CG11943	*D. elegans*	FJ889382
	D. gunungcola	FJ889383
2L		
aristaless	*D. elegans*	FJ889384
	D. gunungcola	FJ889385
timeless	*D. elegans*	FJ889386
	D. gunungcola	FJ889387
echinoid	*D. elegans*	FJ889388
	D. gunungcola	FJ889389

Table S1 (Continued)

Locus	Species	Accession number
arylalkylamine N-acetyltransferase 2	*D. elegans*	FJ889390
	D. gunungcola	FJ889391
Btk family kinase at 29A	*D. elegans*	FJ889392
	D. gunungcola	FJ889393
black	*D. elegans*	FJ889394
	D. gunungcola	FJ889395
yellow c	*D. elegans*	FJ889396
	D. gunungcola	FJ889397
Dopa decarbosylase	*D. elegans*	FJ889398
	D. gunungcola	FJ889399
2R		
Ecdysone Receptor	*D. elegans*	FJ889400
	D. gunungcola	FJ889401
spinster	*D. elegans*	FJ889402
	D. gunungcola	FJ889403
Dopamine Transporter	*D. elegans*	FJ889404
	D. gunungcola	FJ889405
Black cells	*D. elegans*	FJ889406
	D. gunungcola	FJ889407
Dopamine N acetyltransferase	*D. elegans*	FJ889408
	D. gunungcola	FJ889409
3L		
bric a brac 1	*D. elegans*	FJ889410
	D. gunungcola	FJ889411
yellow g	*D. elegans*	FJ889412
	D. gunungcola	FJ889413
pale	*D. elegans*	FJ889414
	D. gunungcola	FJ889415
Clock	*D. elegans*	FJ889416
	D. gunungcola	FJ889417
aracaun	*D. elegans*	FJ889418
	D. gunungcola	FJ889419
yellow k	*D. elegans*	FJ889420
	D. gunungcola	FJ889421

Table S1 (Continued)

Locus	Species	Accession number
Baldspot	*D. elegans*	FJ889422
	D. gunungcola	FJ889423
3R		
yellow e	*D. elegans*	FJ889424
	D. gunungcola	FJ889425
Dopamine Receptor (1)	*D. elegans*	FJ889426
	D. gunungcola	FJ889427
fruitless	*D. elegans*	FJ889428
	D. gunungcola	FJ889429
Hairless	*D. elegans*	FJ889430
	D. gunungcola	FJ889431
ebony	*D. elegans*	FJ889432
	D. gunungcola	FJ889433
torso-like	*D. elegans*	FJ889434
	D. gunungcola	FJ889435
TfIIA-L	*D. elegans*	FJ889436
	D. gunungcola	FJ889437
Dopamine Receptor2	*D. elegans*	FJ889438
	D. gunungcola	FJ889439
4		
cubitus interruptus	*D. elegans*	FJ889440
	D. gunungcola	FJ889441

[#] The chromosome arm where loci reside in *D. melanogaster*.

*DNA sequence information between left and right primers of *dusky* is incomplete. Thus, the sequences obtained for designing the primer set of *dusky* are submitted separately.
